# Aspergillus species and gut microbiota-mediated biotransformation of epigallocatechin gallate: From dark tea fermentation to intestinal metabolism

**DOI:** 10.1016/j.crfs.2026.101587

**Published:** 2026-10-08

**Authors:** Zexin An, Rongrong Ma, Xiangxiang Huang, Yuhang Zhang, Jianan Huang, Zhonghua Liu, Kunbo Wang, Mingzhi Zhu

**Affiliations:** a Key Laboratory of Tea Science of Ministry of Education, Hunan Agricultural University, Changsha, 410128, China; b Yuelushan Laboratory & National Key Laboratory for Tea Plant Germplasm Innovation and Resource Utilization, Changsha, 410128, China; c National Research Center of Engineering and Technology for Utilization of Botanical Functional Ingredients, Hunan Agricultural University, Changsha, 410128, China; d Key Laboratory for Evaluation and Utilization of Gene Resources of Horticultural Crops, Ministry of Agriculture and Rural Affairs of China, Hunan Agricultural University, Changsha, 410128, China; e College of Horticulture, Fujian Agriculture and Forestry University, Fuzhou, Fujian, 350002, China

**Keywords:** Epigallocatechin gallate, *Aspergillus cristatus*, *Aspergillus niger*, Biotransformation, Dark tea, Gut microbiota

## Abstract

Epigallocatechin gallate (EGCG) is the most abundant and bioactive compound in tea, but its microbial biotransformation and mechanisms *in vitro* and in vivo remain unclear. Here, we investigated metabolisms of EGCG and dark tea by *Aspergillus cristatus* and *A. niger*, alongside EGCG biotransformation in rat intestines and *in vitro* gut microbiota. Epigallocatechin-(4β→8)-epicatechin 3,3′-digallate, epigallocatechin, oolongtheanin, and theasinensin B were among the EGCG-associated metabolites detected during fermentation by both fungi, whereas samarangenin A was detected as an early-stage EGCG-associated metabolite in *A. cristatus* fermentation. 3-Hydroxyflavone is a major late-stage EGCG-associated metabolite in *A. cristatus*, while *A. niger* produces a more complex array of metabolites. In liquid culture, *A. cristatus* showed faster EGCG disappearance than *A. niger*, whereas *A. niger* showed faster EGCG depletion in dark tea, indicating a substrate-dependent difference between the two fungi. The key intestinal transit window for EGCG in rats is 6-12 h post-administration. *In vitro*, gut microbiota converted EGCG to monomeric catechins, while several sulfated catechin-related features were also detected. 16S rDNA sequencing revealed temporal changes in the relative abundances of dominant genera, including Ligilactobacillus, Lactobacillus, Streptococcus, and Escherichia-Shigella. This study lays the groundwork for optimizing dark tea fermentation and EGCG functional product development.

## Highlights

• The in vivo metabolic pathways of EGCG were investigated through non-targeted metabolomics.
• Several downstream metabolites associated with EGCG were identified.
• Metabolic transformation information of dark tea fermented by *A. cristatus* and *A. niger* has been supplemented.

## Graphical abstract

**Figure:**
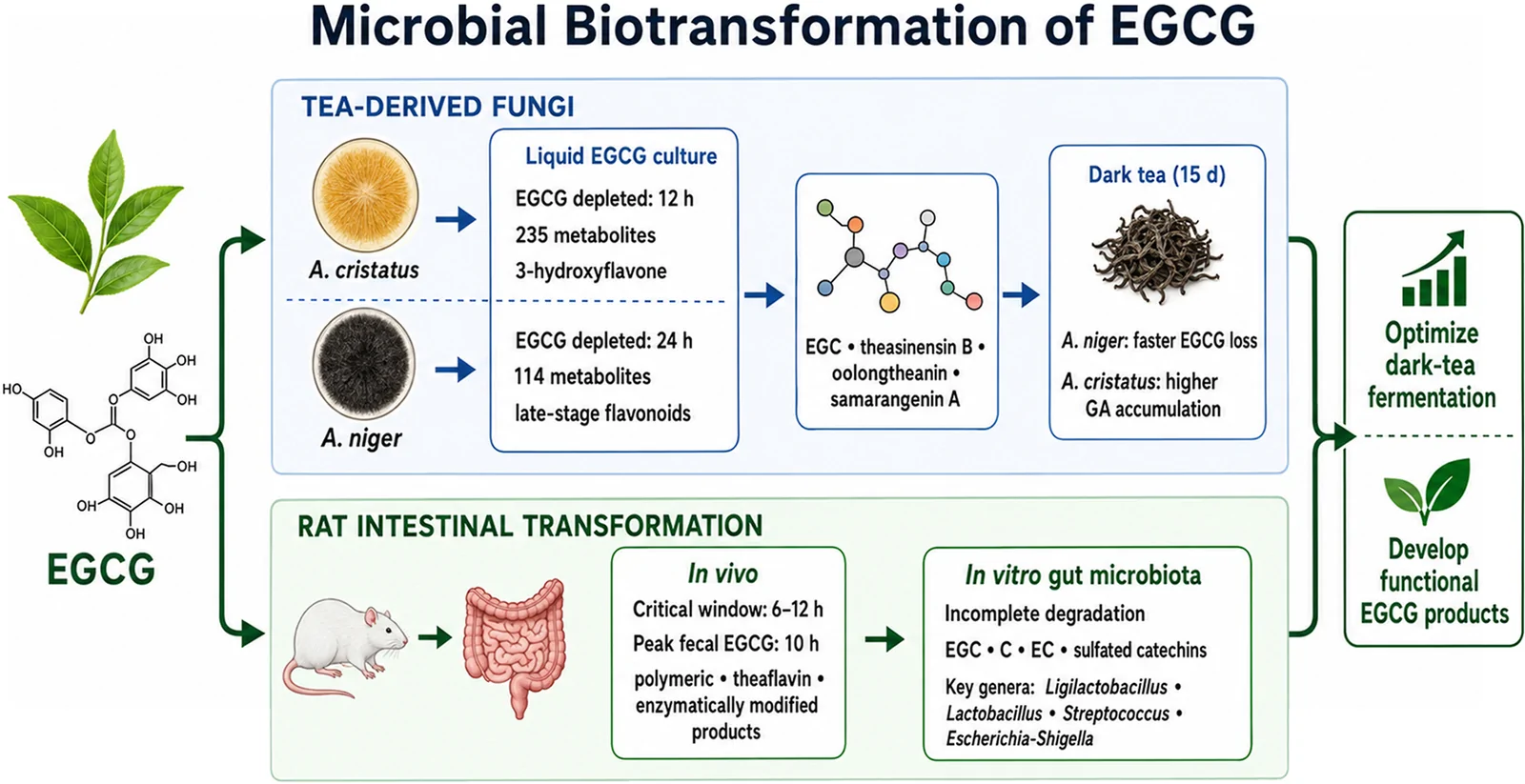

## 1 Introduction

Epigallocatechin gallate (EGCG), a major bioactive catechin in fresh tea leaves, accounts for approximately 50% of total catechins and exhibits antioxidant, anti-inflammatory, and anticancer functions (Meng et al., 2002). During tea fermentation or post-fermentation, EGCG undergoes enzymatic and microbial transformations, resulting in derivatives such as epigallocatechin (EGC), epicatechin (EC), catechin (C), theasinensins, theaflavins, fuzhuanins, and teadenols. These derivatives often exhibit enhanced bioavailability and diverse biological activities (Butt et al., 2014; T. Liu et al., 2023; Sang et al., 2005; Xie et al., 2020). Recently, many studies have found that microbial biotransformation, particularly by fungi and gut microbiota, has been identified as a conversion method with relatively high bioavailability and stability for catechins (Lai et al., 2020; C. Liu et al., 2024; T. Liu et al., 2023).

Dark tea, a unique post-fermented tea produced through solid-state microbial fermentation, is rich in pharmacological properties, such as lipid-lowering and antidiabetic effects (Huang et al., 2024; Xiang et al., 2021; Ye et al., 2024). *Aspergillus cristatus* (also named *Eurotium cristatum*) and *Aspergillus niger* play a crucial role in the fermentation process of dark tea. They influence the synthesis of tea leaf metabolites by secreting extracellular enzymes (Zhu et al., 2020). It is noteworthy that these two fungi play a pivotal role in the biotransformation of catechins in dark teas (Cheng et al., 2025; Q. Zhang et al., 2024). Past studies have shown that, in dark tea fermented solely by *A. cristatus*, EGCG, non-galloylated catechins and gallic acid (GA) levels decrease during prolonged fermentation, accompanied by extensive flavonoid biotransformation (An et al., 2021; Xiao et al., 2022). Additionally, in dark tea or EGCG-based media fermented solely by *A*. *niger*, its ability to effectively degrade EGCG has been observed (Ni et al., 2015; Sun et al., 2024). To date, most research has focused on the conversion of EGCG into EGC and GA, with limited attention paid to other metabolites (Zhang et al., 2024). Therefore, studying the EGCG biotransformation process mediated by *A. cristatus* and *A. niger*, as well as their metabolic transformation patterns on tea substrates, is of significant interest.

The absorption and metabolism of catechins in the body are closely associated with the gut microbiota. Only a small portion of catechins is directly absorbed in the small intestine, while the majority reaches the colon, where it is degraded by gut microbiota. Some metabolites and unmetabolized catechins are reabsorbed via enterohepatic circulation, while most are excreted in feces (Chen and Yang, 2020; Liu et al., 2024). The biotransformation process of EGCG in the intestine has been reported. EGCG is first degraded into EGC and GA by gut microbiota and subsequently converted into various metabolites, including hydroxybenzene isopropanol, phenyl-γ-valerolactones, their related phenylvaleric acids, and phenolic acids by different microbial species. These metabolites exhibit diverse biological activities, such as cytoprotective, anticancer, antioxidative, antidiabetic, and hypotensive effects (Zhang et al., 2024). This process enhances the bioavailability of catechins and diversifies their biological activities (Liu et al., 2024). However, the interactions between EGCG and its derivatives remain poorly understood.

In this study, we integrated targeted and untargeted metabolomics to investigate the spatiotemporal biotransformation of EGCG in separate single-strain fermentation systems using *A. cristatus* and *A. niger.* We further identified core EGCG metabolites in dark tea fermented solely by *A. cristatus* and *A. niger*, analyzed its biotransformation flux in the rat gut, and explored *in vitro* transformations by rat gut microbiota. This work aims to deepen our understanding of EGCG biotransformation under tea-derived fungi and gut microbiota fermentation and facilitate the discovery of novel derivatives with enhanced bioavailability and biological functions.

## 2 Materials and methods

### 2.1 Fungi strain and cultivation

Fu brick tea-derived *A. cristatus* (CCTCC accession number: M20211325) and Liubao tea-derived *A. niger* were used as fungi materials in this work. The fungi were cultivated at 28°C in potato dextrose agar (PDA, Guangdong Huankai Microbial Technology Co., Ltd, Guangzhou, China) for five days. The conidia were then harvested and suspended in sterile water. Concentration of the spore suspensions was adjusted to 5 × 10^7^ spores/mL.

### 2.2 Separate single-strain fermentation of EGCG with *A. cristatus* and *A. niger*

For the two fungal fermentation systems, 1 mL of *A. cristatus* or *A. niger* spore suspension was separately inoculated into the liquid medium containing 0.5 g NaCl, 1 g peptone, and 100 mL sterile distilled water. Thus, *A. cristatus* and *A. niger* were cultured in separate single-strain fermentation systems and were not co-cultured. The cultures were incubated at 28°C with shaking at 180 rpm/min for 5 days, followed by the addition of EGCG to reach a final concentration of 1 mg/mL. Samples were collected from the fermentation broth at 0, 2, 4, 8, 12, 24, 36, and 72 h after EGCG addition. The *A. cristatus* fermentation systems with or without EGCG were designated as the ACF and CK_ACF groups, respectively. Similarly, the *A. niger* fermentation systems with or without EGCG were designated as the ANF and CK_ANF groups, respectively, while EGCG incubated in the sterile medium without fungal inoculation was designated as the CK group. The collected fermentation broth was centrifuged at 10,000 rpm for 10 min at 4°C, and the supernatant was stored at −80°C. The frozen samples were then subjected to freeze-drying. The lyophilized samples were reconstituted in 70% methanol and filtered through a 0.22 μm organic membrane. EGCG and GA were quantified by UPLC at 0, 2, 4, 8, 12, and 24 h, whereas samples collected across the full fermentation time course were analyzed by untargeted metabolomics.

### 2.3 Separate single-strain fermentation of raw dark tea with *A. cristatus* and *A. niger*

The raw dark tea used in this experiment was grade IV material, provided by Anhua Yiyang Tea Factory (Anhua, Yiyang, China). Fifty grams of raw dark tea were mixed with 20 mL distilled water, sterilized at 121°C for 15 min, and cooled. For the fungal fermentation groups, 1 mL of *A. cristatus* or *A. niger* spore suspension was separately inoculated into the sterile tea samples, which were designated as ACFT and ANFT, respectively. In parallel, uninoculated sterile tea samples were maintained under the same fermentation conditions and designated as the control tea group (CKT). The CKT samples underwent the same sterilization and incubation procedures as the inoculated groups and therefore served as a post-autoclaving matrix control for evaluating fungal-associated metabolic changes. All groups were incubated at 28°C for 15 days, and samples were collected in parallel on days 5, 10, and 15. The tea samples were dried at 60°C for 2 h, then ground into a fine powder. A 0.6 g portion was extracted with 10 mL of 70% methanol using ultrasonic treatment for 45 min. The extract was centrifuged at 12,000 rpm for 5 min at 4°C, and the supernatant was filtered through a 0.22 μm organic membrane for targeted and untargeted metabolomics analysis.

### 2.4 Metabolic transformation of EGCG by rat gut microbiota in vivo

EGCG Administration in Rats: Seven-week-old male SPF-grade SD rats (220–250 g) were used for the experiment, fed a standard rodent maintenance diet, and purchased from Changsha Slack Jingda Experimental Animal Co., Ltd. (Changsha, Hunan, China). The study was approved by the Biomedical Research Ethics Committee of Hunan Agricultural University (Approval No. 2021-057). Rats were housed at 25 ± 1°C under a 12-h light/dark cycle. Twelve SD rats were randomly assigned to four subgroups of three animals each. One subgroup received saline and served as the control group (CK, n = 3), whereas the other three subgroups received the same EGCG treatment and were combined for subsequent metabolomics analysis (RE, total n = 9). Because the primary aim of the in vivo experiment was to characterize the temporal metabolic profile following EGCG administration, the larger EGCG-treated cohort provided more biologically independent observations across the sampling time course and broader representation of inter-individual metabolic variation. The saline-treated group was used primarily as a background reference for the fecal metabolome. After one week of acclimation, EGCG treatment was initiated. Rats were fasted for 12 h (with water available) prior to gavage. EGCG was administered as an aqueous solution at a dose of 500 mg kg^−1^ body weight (1.25 mL per rat), while the CK group received an equivalent volume of saline. After gavage, rats were allowed free access to food. Fresh fecal samples were collected at 0, 1, 2, 3, 4, 6, 8, 10, 12, and 24 h using abdominal massage and stored at −80°C.

Fecal Sample Preparation: A 60 mg fecal sample was placed in a sterile 1.5 mL EP tube with two small steel balls. Then, 20 μL of an internal standard (L-2-chlorophenylalanine, Aladdin Industrial Corporation, 0.3 mg/mL; 0.3 mg/mL) and 600 μL of 80% methanol were added for grinding. The mixture was ultrasonicated in an ice-water bath for 10 min and left to stand at −20°C for 30 min. After centrifugation at 13,000 rpm for 10 min at 4°C, 200 μL of the supernatant was dried under a nitrogen stream. The residue was reconstituted in 300 μL of 80% methanol, vortexed for 30 s, ultrasonicated for 3 min, and left to stand at −20°C for 2 h. The mixture was then centrifuged again at 13,000 rpm for 10 min at 4°C, and the supernatant filtered through a 0.22 μm organic membrane into an autosampler vial for analysis.

### 2.5 Metabolic transformation of EGCG by rat gut microbiota *in vitro*

Rat Gut Microbiota Culture: Fresh rat feces (3.0 g) were suspended in 9 mL of physiological saline in a 50 mL centrifuge tube and shaken at 180 rpm for 30 min. The suspension was centrifuged at 5000 rpm for 10 min at 4°C, and the supernatant was collected to obtain the gut microbiota solution. General anaerobic medium, vitamin K1, and hematin chloride were purchased from Qingdao High-Tech Park Haibo Biotechnology Co., Ltd. (Qingdao, Shandong, China) and prepared into anaerobic medium according to the manufacturer’s instructions. The gut microbiota solution was mixed with the anaerobic medium at a 1:9 (v/v) ratio to form the microbiota incubation solution, which was deoxygenated in an anaerobic workstation (HYQX-III, Shanghai Yuejin Medicial Instruments Co., Ltd, Shanghai, China) under 10% CO_2_, 10% H_2_, and 80% N_2_.

Anaerobic fermentation of Gut Microbiota Solution with EGCG (AFE): A total of 100 mL of the prepared gut microbiota incubation solution was divided into 10 groups, with EGCG added to achieve a final concentration of 1.0 mg/mL. The cultures were incubated at 37°C in an anaerobic incubator. Samples were collected at 0, 2, 4, 8, 12, 24, 36, 48, 60, and 72 h, centrifuged at 10,000 rpm for 10 min at 4°C, and the supernatant was stored at −80°C. Control groups were set at 0 h and 72 h, including an EGCG with medium control (CKE) and a anaerobic fermentation of gut microbiota solution control (AFCK). Additionally, anaerobic fermentation of the gut microbiota solution were also conducted using EGCG at final concentrations of 0.5 mg/mL and 0.1 mg/mL, following the same protocol but extending the sampling time points to 0, 4, 8, 12, 24, 48, 72, 96, and 120 h. Control groups (CKE and AFCK) were similarly established at 0 h and 120 h.

Sample Preparation for Metabolomics: The anaerobic fermentation samples were freeze-dried and reconstituted in 300 μL of 80% methanol. After vortexing for 30 s and ultrasonication for 3 min, 20 μL of an internal standard (L-2-chlorophenylalanine, 0.3 mg/mL) was added. The mixture was left at −20°C for 2 h, centrifuged at 13,000 rpm for 10 min at 4°C, and the supernatant filtered through a 0.22 μm organic membrane into an autosampler vial for analysis.

### 2.6 Ultra-performance liquid chromatography (UPLC) analysis

The contents of EGCG and GA were measured using a Waters ACQUITY UPLC system (Waters, Milford, MA, USA) equipped with an HSS T3 column (2.1 mm × 100 mm, 1.8 μm; Waters, Milford, MA, USA). Detection was performed at 278 nm with a 10 μL injection volume, a column temperature of 40°C, and a flow rate of 0.3 mL min^−1^. The mobile phase consisted of ultrapure water (A) and acetonitrile (B), with a gradient elution starting at 10% B, increasing to 17% over 6 min, held for 2.5 min, then reduced to 10% at 8.5 min and maintained until 10 min. EGCG and GA standards (purity ≥ 98%) were purchased from Solarbio Technology Co., Ltd. (Beijing, China), and all other reagents were obtained from Sinopharm Chemical Reagent Co., Ltd. (Beijing, China). UPLC-derived quantitative results were reported according to sample matrix: μg/mL for liquid fermentation samples and μg/g for dark-tea and fecal samples. For comparisons between fungal treatments, EGCG disappearance was evaluated based on the decrease in residual EGCG concentration over time relative to the corresponding initial level.

### 2.7 Untargeted metabolomics analysis

Untargeted metabolomics analysis was performed using an UltiMate 3000 ultrahigh-performance liquid chromatography system (Thermo Fisher Scientific, Waltham, MA, USA) coupled with a Q-Exactive Orbitrap mass spectrometer (Thermo Fisher Scientific, Waltham, MA, USA). Chromatographic separation was performed on a Waters ACQUITY UPLC HSS T3 column (2.1 × 100 mm, 1.8 μm; Waters, Milford, MA, USA) at a flow rate of 0.3 mL/min. The mobile phases consisted of water containing 0.01% formic acid (A) and acetonitrile containing 0.01% formic acid (B). The gradient program was as follows: 0–5 min, 98–95% A; 5–20 min, 95–80% A; 20–25 min, 80–65% A; 25–28 min, 65–0% A; and 28–30 min, 0% A. The column temperature was maintained at 45°C, and the injection volume was 5 μL.

MS data were acquired in data-independent acquisition (DIA) mode using electrospray ionization in both positive and negative ion modes. The MS and MS/MS scan ranges were m/z 100–1200 and m/z 50–1200, respectively. Collision energies of 35 eV and −35 eV were applied in the positive and negative ion modes, respectively, with a collision-energy spread of 15 eV. Auto-calibration was performed between analyses, and each sample was analyzed in triplicate.

Raw LC–MS data were processed using Progenesis QI software (Waters, Milford, MA, USA). Chromatographic alignment, peak picking and integration, adduct deconvolution, and feature annotation were performed using Progenesis QI. Features with more than 50% missing values within a group were removed, and zero values were replaced with half of the minimum detected value. Metabolite annotations with a Progenesis QI identification score below 36 (maximum score, 60) were excluded before construction of the annotated feature matrix. The precursor-ion and product-ion mass tolerances were set at 5 and 10 ppm, respectively, and a product-ion threshold of 5% was applied for MS/MS-based annotation. Metabolites were annotated by matching accurate mass and MS/MS information against the Human Metabolome Database (HMDB), LIPID MAPS database (v2.3), METLIN database, and an in-house database. For the proposed EGCG biotransformation pathways, the evidence supporting each connection was further classified as accurate mass (AM), MS/MS fragmentation (MS2), authentic standard (STD), literature precedent (LIT), GNPS molecular networking (GNPS), or temporal correlation (TC), as summarized in Table S13. The resulting metabolomic data were subsequently subjected to multivariate statistical analysis.

### 2.8 Microbiota sequencing of rat gut microbiota incubation solution

16S rDNA sequencing of AFE (1.0 mg/mL) samples at different fermentation times was performed using the NovaSeq 6000 system. Sequencing was conducted by Novogene Co., Ltd. (Beijing, China). Detailed methods are described in a previous publication (Huang et al., 2024). Given the taxonomic resolution of the 16S rDNA sequencing data, microbial community composition was primarily characterized at the genus level, with emphasis on temporal changes in the relative abundances of major bacterial genera during fermentation.

### 2.9 Statistical analysis

The replicate number and experimental unit were defined separately for each experimental system. The fungal liquid-fermentation experiments included three independent fermentation cultures per treatment (n = 3). For the dark-tea fermentation experiments, one raw-tea source batch was divided into five portions and independently fermented, constituting five independent fermentation units per treatment (n = 5). For the in vivo metabolomics experiment, each individual rat was considered one biological replicate (CK, n = 3; RE, n = 9),whereas fecal samples collected from the same rat at different time points were treated as longitudinal repeated measurements rather than independent biological replicates. For the *in vitro* gut-microbiota experiment, fecal samples were obtained from five rats (donor n = 5), and three parallel anaerobic fermentation cultures were included for each condition/time point (n = 3). Triplicate LC–MS measurements were technical analytical replicates and were not counted as biological replicates.

Data were expressed as the mean ± standard deviation (SD). Statistical comparisons were performed using one-way analysis of variance (ANOVA). For the in vivo longitudinal metabolomics analysis, metabolite intensities were log-transformed as log(1 + x) and analyzed using a linear mixed-effects model, with time included as a fixed effect and rat identity as a random effect to account for within-animal correlations across repeated measurements. P values were adjusted using the Benjamini–Hochberg procedure to control the false discovery rate (FDR). For the other experimental systems, statistical comparisons were performed using one-way analysis of variance (ANOVA). Associations between dominant bacterial genera and EGCG or EGCG-associated metabolites were evaluated using Spearman rank correlation analysis based on matched mean values at the five 16S rDNA sampling time points (0, 12, 24, 48, and 72 h; n = 5). Given the small number of paired time points, two-sided exact permutation P values were calculated. P values from the 40 taxon–metabolite comparisons were adjusted using the Benjamini–Hochberg procedure to control the false discovery rate (FDR), with FDR-adjusted P < 0.05 considered statistically significant.

Principal component analysis (PCA), orthogonal projection to partial least-squares discriminant analysis (OPLS-DA), hierarchical clustering analysis (HCA), and variable importance in projection (VIP) analyses were conducted using SIMCA 14.1 (Umetrics AB, Umea, Sweden). Heatmaps were generated using R 4.2.1 (https://www.r-project.org). Sankey diagrams were visualized with RAWGraphs (https://www.rawgraphs.io). Global Natural Products Social Molecular Networking (GNPS) analysis was performed via the GNPS platform (https://gnps.ucsd.edu/ProteoSAFe/index.jsp). Weighted correlation network analysis (WGCNA), adapted here for metabolite abundance data, was conducted with TBtools (https://github.com/CJ-Chen/TBtools). Pearson correlation was used for correlation analysis, and an unsigned network was constructed using a soft-thresholding power (β) of 26. The minimum module size was set to 10, and modules were merged using a merge cut height of 0.15. For network export, a topological overlap matrix (TOM)-based edge-weight threshold of 0.1 was applied. The resulting metabolite correlation networks were visualized using Cytoscape 3.7.1 (https://cytoscape.org). Different letters within the same graph indicate statistically significant differences (P < 0.05). The apparent disappearance kinetics of EGCG during fungal fermentation were evaluated using a first-order kinetic model, ln(Ct) = ln(C0) − kappt, where Ct is the EGCG concentration at time t and kapp is the apparent first-order disappearance rate constant. The half-life (t1/2) was calculated as ln(2)/kapp. Only time points with quantifiable EGCG concentrations were included in the kinetic regression. The resulting kinetic parameters are summarized in Table S14.

Metabolite screening and prioritization. To clarify the stepwise reduction of the metabolomic datasets, the complete metabolite-screening workflow is summarized in Table S15. The starting feature numbers reported in this study refer to annotated metabolic features retained after data preprocessing and metabolite annotation rather than unprocessed raw LC–MS ion peaks. For the fungal liquid-fermentation experiments, 3402 annotated features were obtained, and differential features were retained using FC > 2 or FC < 0.5 relative to the corresponding controls, resulting in 235 and 114 differential metabolites in ACF and ANF, respectively. Their temporal abundance patterns relative to EGCG and GA were subsequently used to prioritize candidates for the proposed pathways. For dark-tea fermentation, 5144 annotated features were analyzed using WGCNA, and metabolites in EGCG/GA-associated modules were further prioritized using kME > 0.9 and GS > 0.9. For the in vivo rat dataset, annotated metabolite-related features (MRFs) were first screened according to chemical classification to retain structural classes potentially relevant to EGCG biotransformation, including benzoic acids and derivatives, hydrolyzable tannins, and flavonoid-related features. To avoid overlap among hierarchical chemical categories, flavonoid-related MRFs were assigned to mutually exclusive subclasses, with “other flavonoids” defined as flavonoid-related features not classified as flavans, flavones, or isoflavans. The resulting candidate MRFs were subsequently prioritized using VIP > 1 and mixed-effects model-derived FDR-adjusted P < 0.05. Structurally and temporally supported metabolites were further selected for construction of the proposed EGCG biotransformation pathways. For the *in vitro* gut-microbiota experiment, nine flavans were extracted from 5942 annotated AFMs. Thus, differential-metabolite analysis, WGCNA, and VIP filtering were applied as parallel, system-specific prioritization strategies rather than consecutive screening steps.

## 3 Results

### 3.1 Biotransformation of EGCG by *A*. *cristatus* and *A*. *niger*

To understand the mechanisms of tea polyphenol transformation mediated by *A. cristatus* and *A. niger*. UPLC was used to analyze the biotransformation of EGCG by *A. cristatus* and *A. niger* over different fermentation periods (0, 2, 4, 8, 12, and 24 h). In the CK (sterile medium control) group, EGCG and GA levels remained stable during 0–24 h of fermentation, indicating a low rate of EGCG auto-oxidation in the medium (Fig. 1A). EGCG decreased continuously in both fungal systems (Fig. 1B and C). In ACF, EGCG became undetectable by 12 h, whereas it remained detectable at 12 h in ANF and became undetectable by 24 h. First-order kinetic analysis yielded apparent disappearance rate constants (kapp) of 0.326 and 0.107 h−1 for ACF and ANF, respectively, with corresponding half-lives of 2.13 and 6.49 h (Table S14). Thus, the apparent EGCG disappearance rate constant in ACF was approximately 3.05-fold that in ANF. GA generally accumulated during the 0–24 h fermentation period in both systems. Together, these results indicate that the major disappearance of EGCG occurred within the first 24 h of fermentation. Accordingly, the later fermentation stages were interpreted primarily on the basis of temporal changes in EGCG- and GA-associated metabolic features.

**Fig. 1:**
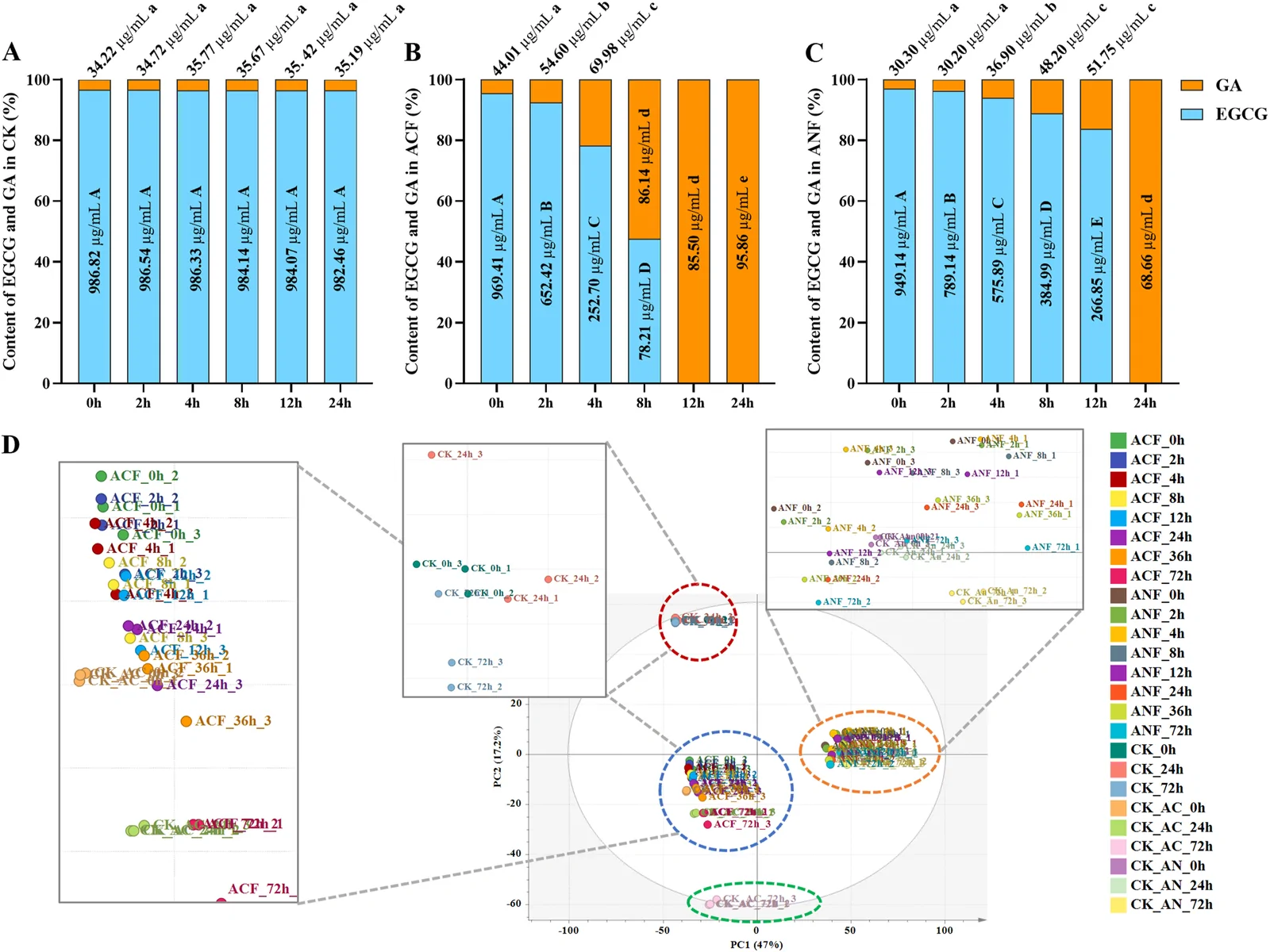
Targeted and untargeted metabolomics of epigallocatechin gallate (EGCG) during separate single-strain fermentation with *Aspergillus cristatus* or *Aspergillus niger* at different time points. (A-C) Stacked bar graphs showing the percentage content of EGCG and gallic acid (GA) in CK (sterile medium control, A), ACF (fermentation with *A. cristatus* and EGCG at 1.0 mg/mL, B), and ANF (fermentation with *A. niger* and EGCG at 1.0 mg/mL, C) detected by ultra-performance liquid chromatography (UPLC) analysis. EGCG and GA concentrations (μg/mL) are added to the bars. Significant differences in concentrations are marked with different uppercase (EGCG) or lowercase (GA) letters (one-way ANOVA; *P* < 0.05). Legends are consistent across panels A-C. (D) Principal component analysis (PCA) plot of the metabolites in CK, CK_ACF (*A*. *cristatus* fermentation control), CK_ANF (*A*. *niger* fermentation control), ACF, and ANF groups at different fermentation times, based on negative ion mode data from ultra-high-performance liquid chromatograph-Q-Exactive Orbitrap tandem mass spectrometry. Dotted ellipses in different colors classify the metabolites in these fermentation samples into four categories.

To comprehensively analyze EGCG biotransformation by *A. cristatus* and *A. niger*, we employed UHPLC-Q-Exactive Orbitrap-MS to examine the metabolic profiles of the separate fungal fermentation systems containing EGCG at various time points (0, 2, 4, 8, 12, 24, 36, and 72 h). The groups were set as follows: CK; CK_AC, *A*. *cristatus* fermentation control; CK_AN, *A*. *niger* fermentation control; ACF; and ANF. A total of 3402 annotated metabolic features were detected in all fermentation solutions in negative-ion mode (Table S1). Principal component analysis (PCA) of 3402 metabolites revealed clear differentiation among the different groups. This result indicates that indicated significant differences in EGCG biotransformation by *A*. *cristatus* and *A*. *niger*. In *A*. *cristatus* fermentation, both fermentation time and the presence of EGCG in the medium significantly influenced the metabolites.

Under microbial influence, compounds undergo chemical reactions such as cleavage, redox, methylation, and hydroxylation, which can affect their bioactivity or stability. OPLS-DA was used to analyze the metabolic transformation patterns of EGCG under *A*. *cristatus* and *A*. *niger* fermentation at different time points. After excluding control-associated changes, features showing a fold change (FC) > 2 or FC < 0.5 relative to the corresponding controls were retained, resulting in 235 and 114 differential metabolites in ACF and ANF, respectively (Tables S2 and S3). The aforementioned metabolites may be produced by EGCG through fermentation by *A*. *cristatus* and *A*. *niger*. In ACF group, clustering heatmap analysis displayed the abundance of these differential metabolites at different fermentation times (Fig. 2A). The abundance of EGCG (Met, 1863) decreased with fermentation time and stabilized after 12 h of fermentation (Table S2), consistent with the UPLC results. Based on the changes in EGCG and GA levels, we categorized the differential metabolites in the clustering heatmap into four groups: EGCG-related early-stage metabolites (red box), mid-stage associated metabolites (green box), late-stage associated metabolites (blue box), and GA-related products (purple box) in ACF. In the red box, the level trends of EGC (Met, 1862), samarangenin A (Met, 1909), and epigallocatechin-(4*β*→8)-epicatechin 3,3′-digallate (Met, 1935) matched that of EGCG, suggesting they were early-stage EGCG-associated metabolites under *A. cristatus* fermentation. Additionally, a group of metabolites negatively correlated with EGCG levels during 0–12 h of ACF (green box) was identified, including morelosin (Met 235), demethyltorosaflavone D (Met 625), theasinensin B (Met, 1944), oolongtheanin (Met, 1899), prodelphinidin A2 3′-gallate (Met, 1921), theasinensin A (Met 377), isoneotheaflavin 3-O-gallate (Met, 1867), and theaflavin 3,3′-digallate (Met, 1904). These metabolites were likely key EGCG-associated metabolites under *A. cristatus* fermentation. Meanwhile, the blue box in Fig. 2A highlighted the potential late-stage EGCG-associated metabolites in ACF. Among these, 3-hydroxyflavone (Met, 1893) was likely a late-stage EGCG-associated metabolite. The left panel of Fig. 2B illustrated the potential metabolic conversion pathway of EGCG during *A. cristatus* fermentation. In addition, numerous compounds potentially associated with GA metabolism were identified in ACF. The right panel of Fig. 2B illustrated the potential metabolic conversion pathway of GA during *A*. *cristatus* fermentation. GA represents the primary metabolite from EGCG degradation. It undergoes enzymatic oxidation, where microbial enzymes selectively remove or rearrange hydroxyl groups on the benzene ring, resulting in the formation of 2,3,4-trihydroxybenzoic acid (Met 28). Cromoglicic acid (Met 1720) was likely a GA-associated metabolite during *A. cristatus* fermentation. Moreover, several late-stage metabolic features showed temporal associations with GA during *A. cristatus* fermentation, including Met 31, Met 39, Met 49, Met 62, Met 371, and Met 1905. Database-assisted annotation provided tentative structural assignments for these features, corresponding to lidoflazine, camonagrel, butralin, pioglitazone, dihydroxyphaseic acid, and ellagic acid, respectively. Fig. 2C illustrated the classification of 235 differentially accumulated metabolites in ACF, providing a foundational framework for understanding the composition of EGCG degradation products by *A. cristatus*.

**Fig. 2:**
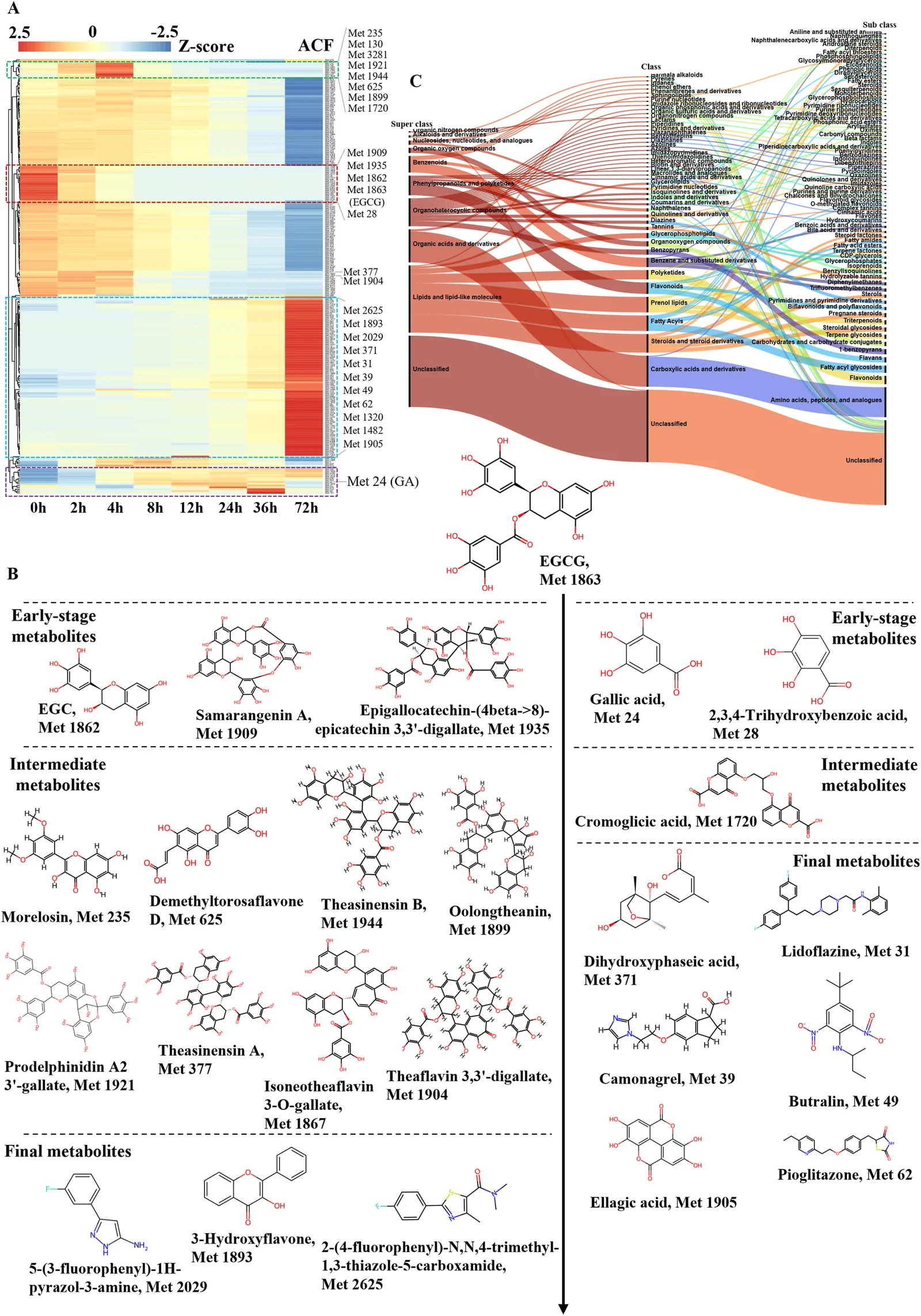
Differential metabolites based on untargeted metabolomics identified during co-fermentation of epigallocatechin gallate (EGCG) and *Aspergillus cristatus* (ACF) at different time points. (A) Heatmap of Z-score normalized levels of differential metabolites in ACF. The red box highlights early-stage EGCG-associated metabolites; the green box highlights mid-stage EGCG-associated metabolites; the blue box highlights late-stage EGCG-associated metabolites; the purple box highlights late-stage GA-associated metabolites. (B) Potential metabolic pathways of EGCG in ACF. The left panel shows proposed EGCG-related transformations, while the right panel shows GA-related transformations. The evidence supporting each proposed connection is summarized in Table S13, including accurate mass, MS/MS fragmentation, authentic standard, literature precedent, Global Natural Products Social (GNPS) molecular networking, and temporal correlation, where applicable. (C) Sankey diagram of the annotation categories for the 235 differential metabolites in ACF. Detailed information on the compounds corresponding to Met ID can be found in Table S1.

The EGCG content in ANF decreased with increasing fermentation time of *A. niger* and stabilized after 24 h. This trend was consistent with the UPLC results. (Fig. 3A). Furthermore, based on the EGCG branch (red box in Fig. 3A), we identified epigallocatechin-(4*β*→8)-epicatechin 3,3′-digallate (Met, 1935) and GA as early-stage EGCG-associated metabolites (Fig. 3B). Based on the EGC branch (green box in Fig. 3A), we identified 29 metabolites as potential mid-stage EGCG-associated metabolites of EGCG during *A. niger* fermentation (0–24 h). These included six flavans (EGC, Met, 1862; isoneotheaflavin 3-O-gallate, Met, 1867; oolongtheanin, Met, 1899; theaflavin-3-gallate, Met, 1868; tragopogonic acid, Met, 1887; and isotheaflavin 3′-gallate, Met, 1890), two biflavonoids and polyflavonoids (prodelphinidin A2 3′-gallate, Met, 1921; and 3′-galloylprodelphinidin B2, Met, 1876), one polyketide (demethyltorosaflavone D, Met 625), and one complex tannin (theasinensin B, Met, 1944). Additionally, the clustering heatmap (blue box in Fig. 3A) revealed numerous potential late-stage (24–36 h) and final-stage (36–72 h) metabolites related to EGCG metabolism, including seven flavonoids. Among them, the relative contents of quercetin 3-(6″-caffeylgalactoside) (Met 227), 5,7,3′,4′,5′-pentahydroxyflavanone (Met 367), isotheaflavin (Met, 1874), fuzhuanins A (Met, 1877), and prodelphinidin A1 (Met, 1892) peaked at 36 h and then decreased by 72 h. In contrast, pelargonidin 3-sambubioside (Met 413) and 7,2′-dihydroxy-4′,5′-methylenedioxy-3-phenylcoumarin (Met 488) reached their highest levels at 72 h, suggesting they may represent the late-stage EGCG-associated metabolites during *A. niger* fermentation. In addition, the early-stage metabolites of GA during *A. niger* fermentation were consistent with those observed in *A. cristatus*. (E)-N-(3-(3-(4-Fluorophenoxy)phenyl)-1-(R,S)-methylprop-2-enyl)-N-hydroxyurea (Met 220) and salicyl-6-hydroxy-2-cyclohexene-on-oyl (Met 37) were identified as potential GA-associated metabolites in ANF. Moreover, theacitrin A (Met 55), gentisic acid (Met 52), 2,2′-methylenebis(4-methyl-6-tert-butylphenol) (Met 25), dehydrochorismic acid (Met 81), Met 2761, and Met 3339 were identified as likely late-stage GA-associated metabolites during *A. niger* fermentation. The potential biotransformation pathways of EGCG and GA during *A. niger* fermentation were depicted in Fig. 3B. Fig. 3C illustrated the classification of the 114 differentially accumulated metabolites associated with EGCG metabolism after *A. niger* fermentation. In summary, the above results reveal EGCG-related downstream metabolites generated after fermentation of *A. niger*. This provides insights into the potential metabolic transformation of EGCG by *A. niger*. The proposed connections in Fig. 2, Fig. 3B are based on different levels of evidence, including accurate mass/MS/MS annotation, temporal changes, literature precedent, and, for selected metabolites, GNPS molecular-networking support. The evidence for each connection is specified in Table S13.

**Fig. 3:**
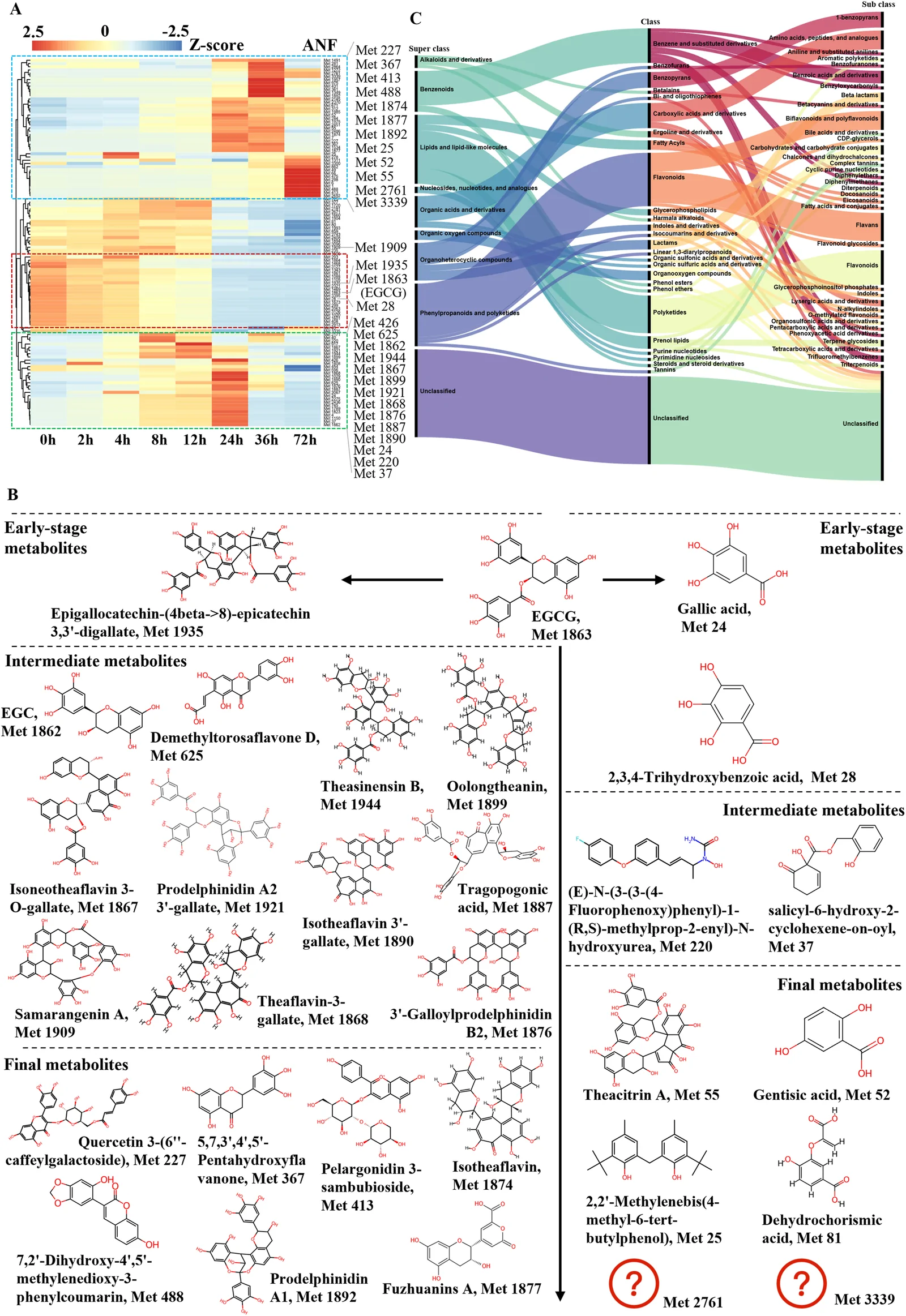
Differential metabolites based on untargeted metabolomics identified during co-fermentation of epigallocatechin gallate (EGCG) and *Aspergillus niger* (ANF) at different time intermediate products points. (A) Heatmap of Z-score normalized levels of differential metabolites in ANF. The red box highlights early-stage EGCG-associated metabolites; the green box highlights mid-stage EGCG-associated metabolites; the blue box highlights late-stage EGCG-associated metabolites. (B) Potential metabolic pathways of EGCG in ANF. The left panel shows proposed EGCG-related transformations, while the right panel shows GA-related transformations. The evidence supporting each proposed connection is summarized in Table S13, including accurate mass, MS/MS fragmentation, authentic standard, literature precedent, GNPS molecular networking, and/or temporal correlation, where applicable. (C) Sankey diagram of the annotation categories for the 114 differential metabolites in ANF. Detailed information on the compounds corresponding to Met ID can be found in Table S1.

### 3.2 EGCG biotransformation in dark tea fermented by *A*. *cristatus* and *A*. *niger*

*A. cristatus* and *A. niger* are dominant fungi in dark tea and play a critical role in tea quality transformation. Typically, the microbial fermentation during the “flowering” stage in Fu brick tea production lasts approximately 15 days. To explore the metabolic transformation of EGCG by *A. cristatus* and *A. niger*, sterile raw dark tea was fermented with these fungi. EGCG and GA contents were analyzed after 5, 10, and 15 days of fermentation. Significant visual differences emerged between dark teas fermented by *A. cristatus* (ACFT) and *A. niger* (ANFT) over time. On day 5, no visible fungal growth was observed in the ACFT group, whereas a small amount of white mycelium appeared in the ANFT group. By day 10, sparse golden particles and mycelium developed in ACFT, while *A. niger* exhibited vigorous growth in ANFT, turning the leaves brownish. By day 15, abundant golden particles were present in ACFT, whereas the tea in ANFT had fully transformed to a brownish color. UPLC analysis revealed that the EGCG content in raw dark tea decreased over time during fermentation with *A. cristatus* (Fig. 4A). During the first 10 days of fermentation, the EGCG content decreased significantly, while between days 10 and 15, the EGCG content remained virtually unchanged. In the ACFT, the abundance trend of GA first decreased and then increased (Fig. 4B). In ANFT, EGCG content dropped to 209.5 μg/g by day 5, showing no significant difference from the levels at day 10, indicating more rapid EGCG disappearance during *A. niger* fermentation than during *A. cristatus* fermentation in dark tea. By day 15, EGCG was nearly undetectable in ANFT (Fig. 4A). Similarly, GA levels in ANFT also exhibited an initial decline followed by an increase; however, the increase in GA content was lower compared to ACFT (Fig. 4B). Overall, in both ACFT and ANFT, the trends in EGCG and GA content were similar, with EGCG showing an overall downward trend while GA exhibited an overall upward trend. However, their metabolic rates differ in ACFT and ANFT. These results indicated that *A. niger* degrades EGCG in tea leaves at a faster rate than *A. cristatus*. In contrast, greater GA accumulation was observed during *A. cristatus* fermentation than during *A. niger* fermentation.

**Fig. 4:**
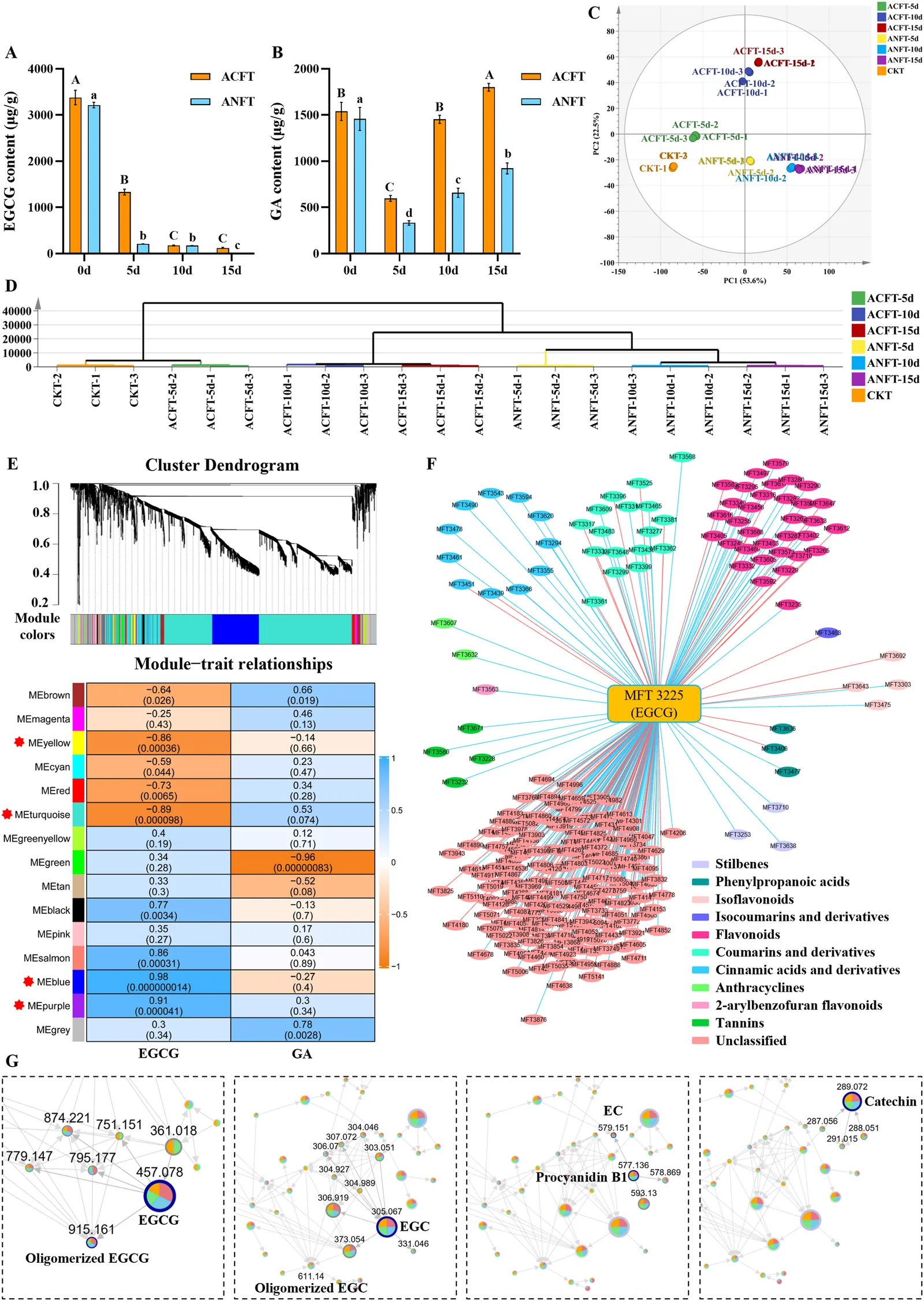
Targeted and untargeted metabolomics of dark tea fermented with *Aspergillus cristatus* (ACFT) and *Aspergillus niger* (ANFT). (A-B) Epigallocatechin gallate (EGCG, A) and gallic acid (GA, B) contents in ACFT and ANFT at different fermentation times detected by ultra-performance liquid chromatography (UPLC) analysis. Significant differences in concentrations are marked with different uppercase (ACFT) or lowercase (ANFT) letters (one-way ANOVA; *P* < 0.05). (C-D) Principal component analysis (PCA) plot (C) and hierarchical cluster analysis (HCA) plot (D) of the metabolites in CKT (control tea group), ACFT, and ANFT groups at different fermentation times, based on negative ion mode data from ultra-high-performance liquid chromatograph-Q-Exactive Orbitrap tandem mass spectrometry (UHPLC-Q-Exactive Orbitrap-MS) analysis. (E) Identification of core metabolite modules by weighted gene co-expression network analysis (WGCNA) in ACFT at different fermentation times based on UHPLC-Q-Exactive Orbitrap-MS analysis. The red heptagrams indicate metabolite modules highly significantly correlated with quantified EGCG content. (F) The co-expression network of potential metabolic relationships between EGCG and 244 metabolites highly associated with it, identified from the Meblue module in ACFT. Orange edges represent positive correlations, while blue edges represent negative correlations. Metabolites with different colors represent different classes. (G) Molecular networks of catechins in ACFT at different fermented times (0, 5, 10, and 15 days) constructed by Global Natural Products Social Molecular Networking (GNPS). Each node represents a compound, with compounds labelled by parent ion mass clustering together, indicating similar chemical structures. Detailed information on the compounds corresponding to MFT ID can be found in Table S4. EGC, epigallocatechin.

Unlike the single-strain fermentation of EGCG, dark tea, rich in secondary metabolites, undergoes highly complex metabolic transformations during fermentation by *A. cristatus* and *A. niger*, involving degradation, oxidation, polymerization, and competitive interactions among metabolites. To elucidate the chemical changes in raw dark tea during fermentation by *A. cristatus* and *A. niger*, as well as the potential metabolic pathways of EGCG, untargeted metabolomics was performed on ACFT and ANFT. A total of 5144 compounds were detected in the control group (CKT), ACFT, and ANFT at days 5, 10, and 15 (ACFT-5d, ACFT-10d, ACFT-15d; ANFT-5d, ANFT-10d, ANFT-15d) by negative ion mode (Table S4). PCA and HCA clustering analyses indicate that some samples are well distinguished. Notably, ANFT-10d and ANFT-15d cluster together, while ANFT-5d is assigned to a separate cluster. This suggests that metabolite conversion rates are higher during the early fermentation stage of ANFT (Fig. 4C and D).

To identify key metabolites in dark tea fermented by *A. cristatus* and *A. niger*, WGCNA was employed to partition 5144 compounds into co-expression modules. Within the ACFT, WGCNA classified these metabolites into 15 distinct color-coded modules (Fig. 4E). Further correlation analysis was conducted between the abundance trends of EGCG and GA in ACFT and metabolic modules. Results revealed that the Meblue module (651 metabolites) and Mepurple module (32 metabolites) showed highly significant positive correlations with EGCG content. In contrast, the Meturquoise (2238 metabolites) and Meyellow (96 metabolites) modules exhibited a highly significant negative correlation with EGCG content. The Megrey (328 metabolites) module showed a highly significant positive correlation with GA content, while the Megreen (77 metabolites) module exhibited a highly significant negative correlation with GA content. In the Meblue module, 585 core metabolites were identified through threshold screening (kME value > 0.9 and GS value > 0.9), including 13 flavans. Among these, core flavans associated with EGCG metabolism in dark tea during *A. cristatus* fermentation included (±)-catechin (MFT 3211), epigallocatechin (MFT 3214), (−)-epiafzelechin 3-gallate (MFT 3255), (±)-taxifolin (MFT 3265), epigallocatechin 3-O-caffeate (MFT 3287), epiafzelechin 3-O-gallate (MFT 3316), taxifolin (MFT 3332), neotheaflavin (MFT 3534), epicatechin 3-O-(4-methylgallate) (MFT 3617), butin (MFT 3647), and (−)-epigallocatechin 3-(3-methyl-gallate) (MFT 3688). Additionally, four core flavans associated with EGCG metabolism were identified in the Meturquoise module: isoneotheaflavin 3-O-gallate (MFT 3236), theaflavic acid (MFT 3261), trans-3,3′,4′,5,5′,7-hexahydroxyflavanone (MFT 3398), and (−)-epigallocatechin 3,3′-di-gallate (MFT 3621). A co-expression metabolic network related to the metabolism of EGCG (MFT 3225) was constructed for the Meblue module using Cytoscape (Fig. 4F). This analysis revealed potential metabolic relationships between EGCG and 244 metabolites highly associated with EGCG. Additionally, we identified 53 metabolites highly correlated with GA in the Megrey and Megreen modules (Table S6). These findings provide a data foundation for understanding the metabolic transformation mechanisms of EGCG and GA in dark tea fermented with *A. cristatus*.

Additionally, a molecular metabolic network of dark tea fermented by *A. cristatus* was constructed using GNPS (Fig. 4G). By clustering structurally similar compounds, several EGCG-related metabolic derivatives were identified in ACFT. These compounds shared a common fragment ion of *m/z* 170.02, with some also exhibiting fragment ions of *m/z* 125.02 or *m/z* 137.02. These compounds were likely EGCG complexes with other molecules, indicating potential upstream and downstream metabolic relationships. Among these, the compound with a parent mass at *m/z* 915.161 is likely an EGCG dimer. Additionally, several compounds were identified with close metabolic relationships to EGC (parent mass: *m/z* 305.067), including a compound with a parent mass of *m/z* 611.14, which is potentially a dimer of (−)-epigallocatechin. Furthermore, procyanidin B1 (parent mass: *m/z* 577.136) may serve as an upstream metabolite of (−)-epicatechin (parent mass: *m/z* 579.151). Catechin (parent mass: *m/z* 289.072) exhibited strong associations with compounds having parent masses of *m/z* 287.056, *m/z* 291.015, and *m/z* 288.051.

In ANFT, WGCNA categorized all metabolites into 11 distinct color-coded modules (Fig. 5A). Among these, the Meblue module (945 metabolites) showed a highly significant positive correlation with both EGCG and GA contents. In contrast, the Mebrown module (378 metabolites) exhibited a highly significant negative correlation with EGCG and GA contents. A total of 900 core metabolites were identified from the Meblue and Mebrown modules (kME value > 0.9 and GS value > 0.9), including 19 flavans (Table S7). Similar to the ACFT group, key flavans associated with EGCG metabolism included theaflavic acid (MFT 3261), epiafzelechin 3-O-gallate (MFT 3316), (−)-epiafzelechin 3-gallate (MFT 3255), (−)-epigallocatechin 3,3′-di-gallate (MFT 3621), trans-3,3′,4′,5,5′,7-hexahydroxyflavanone (MFT 3398), epigallocatechin 3-O-caffeate (MFT 3287), neotheaflavin (MFT 3534), epicatechin 3-O-(4-methylgallate) (MFT 3617), and (−)-epigallocatechin 3-(3-methyl-gallate) (MFT 3688). In addition, several unique core metabolites related to EGCG were identified in ANFT, such as theaflavate B (MFT 3234), epicatechin 3-O-(3-O-methylgallate) (MFT 3347), (±)-taxifolin (MFT 3516), epicatechin (MFT 3238), epiafzelechin (2R,3R)(−) (MFT 3260), 4-gallocatechol (MFT 3340), (−)-epigallocatechin 3-(4-methyl-gallate) (MFT 3440), and 8,8′-methylenebiscatechin (MFT 3606). These differences highlight the variations in flavan metabolism during fermentation by *A. cristatus* and *A. niger* in dark tea. Among these, 4-gallocatechol was significantly accumulated after 15 days of *A. niger* fermentation and may be one of the core late-stage EGCG-associated metabolites. Furthermore, a large number of compounds potentially related to GA metabolism were identified in both the MEblue and MEbrown modules (Table S8). A co-expression network of 353 metabolites highly associated with EGCG (MFT 3225) in the Meblue module was constructed using Cytoscape (Fig. 5B). Among these, EGCG showed potential metabolic relationships with 67 flavonoids. These findings provide a valuable data foundation for understanding the metabolic transformation patterns of dark tea under *A. niger* fermentation.

**Fig. 5:**
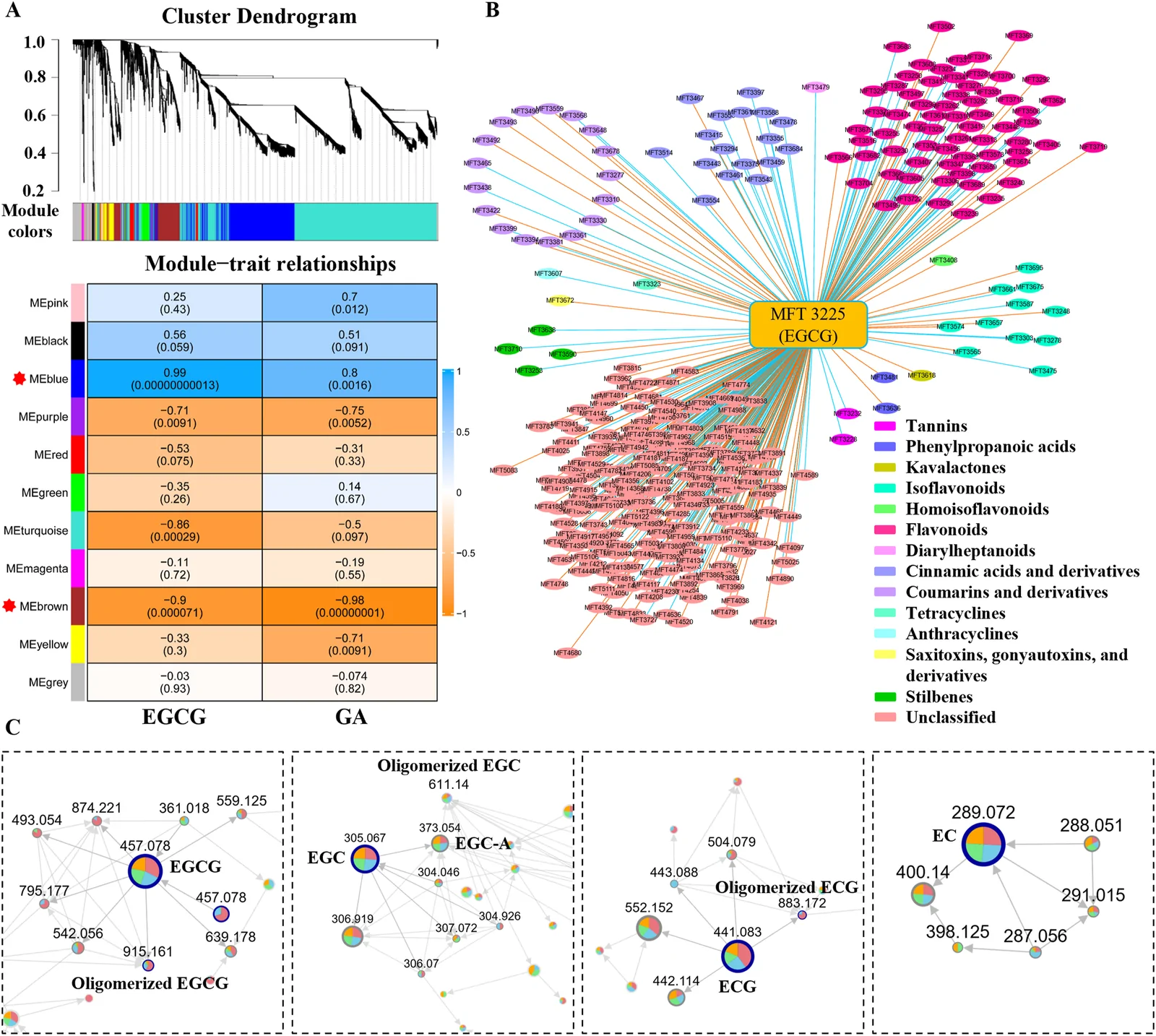
Untargeted metabolomics of dark tea during co-fermentation with *Aspergillus niger* (ANFT). (A) Identification of core metabolite modules by weighted gene co-expression network analysis (WGCNA) in ANFT at different fermentation times (0, 5, 10, and 15 days) based on negative ion mode data from ultra-high-performance liquid chromatograph-Q-Exactive Orbitrap tandem mass spectrometry (UHPLC-Q-Exactive Orbitrap-MS) analysis. The red heptagrams indicate metabolite modules highly significantly correlated with quantified epigallocatechin gallate (EGCG) content. (B) The co-expression network of potential metabolic relationships between EGCG and 353 metabolites highly associated with it, identified from the Meblue module in ANFT. Orange edges represent positive correlations, while blue edges represent negative correlations. Metabolites with different colors represent different classes. (C) Molecular networks of catechins in ANFT at different fermented times constructed by Global Natural Products Social Molecular Networking (GNPS). Each node represents a compound, with compounds labelled by parent ion mass clustering together, indicating similar chemical structures. Detailed information on the compounds corresponding to MFT ID can be found in Table S4. EGC, epigallocatechin; ECG, epicatechin gallate; EC, epicatechin.

By constructing a GNPS molecular network, numerous compounds with structures similar to EGCG, EGC, ECG, and EC were identified in ANFT. These compounds clustered together and exhibited the same fragment ion characteristics. Based on the variation in parent mass and fragment ion, the potential chemical modifications of these compounds can be inferred (Fig. 5C). For example, EGC-A, with a parent ion mass of m/z 373.054, is likely a hydroxymethylated derivative of EGC, indicated by a 68 Da mass increase and characteristic fragmentation patterns of EGC. This hypothesis can be further verified by high-resolution mass spectrometry to determine its molecular formula.

### 3.3 Metabolic conversion of EGCG in vivo

EGCG undergoes extensive metabolic transformation in vivo, which plays a critical role in determining its bioavailability and physiological effects. To analyze the metabolic conversion of EGCG by intestinal microbiota, we administered an EGCG solution to healthy SD rats via gavage. UPLC was used to detect the EGCG content in rat fecal samples collected at various time points (0, 1, 2, 3, 4, 6, 8, 10, 12, and 24 h). Results showed that EGCG was first detected in feces at 6 h post-gavage (169.28 μg/g) (Fig. 6A). Its content gradually increased during the 6–10 h period, reaching a peak of 440.27 μg/g at 10 h. Afterward, the EGCG level began to decrease, with only 6.14 μg/g remaining in the feces at 24 h. This result suggested that the 6–12 h period following EGCG administration appears to be a key intestinal transit window for EGCG in the gut.

**Fig. 6:**
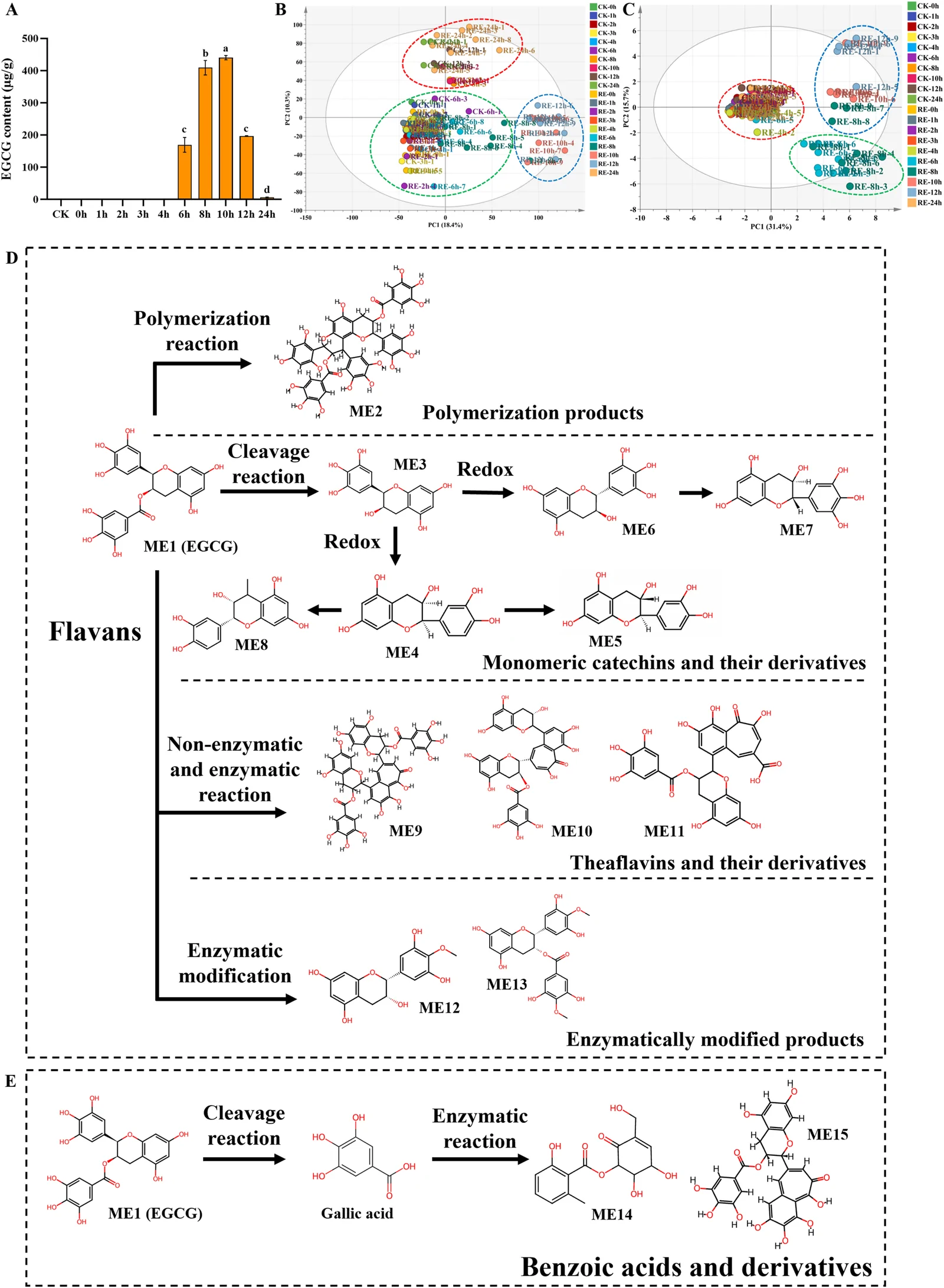
Metabolic conversion of epigallocatechin gallate (EGCG) in rat. (A) EGCG content in rat feces at different time points detected by ultra-performance liquid chromatography (UPLC) analysis. Significant differences in concentrations are marked with different letters (one-way ANOVA; *P* < 0.05). (B) Principal component analysis (PCA) plot of the metabolites in CK (saline control) and RE (EGCG gavage) groups at different time points, based on both positive and negative ion mode data from ultra-high-performance liquid chromatograph-Q-Exactive Orbitrap tandem mass spectrometry (UHPLC-Q-Exactive Orbitrap-MS) analysis. Dotted ellipses in different colors classify the metabolites in these fecal samples into three categories. (C) PCA plot of the 683 EGCG-associated candidate metabolite-related features (MRFs) selected from the complete untargeted metabolomic dataset by chemical-class filtering, including 167 benzoic acids and derivatives, 6 hydrolyzable tannins, and 510 flavonoid-related MRFs comprising 43 flavans, 12 flavones, 9 isoflavans, and 446 other flavonoids. (D) Potential transformation pathways of EGCG-related flavans in the rat intestine. These flavans are selected based on orthogonal projection to partial least-squares discriminant analysis (OPLS-DA) and variable importance in projection (VIP) analyses (VIP > 1 and mixed-effects model-derived FDR-adjusted *P* < 0.05). (E) Potential transformation pathways of gallic acid-related benzoic acids and derivatives in the rat intestine. The evidence supporting each proposed connection in panels D and E is summarized in Table S13, including accurate mass, MS/MS fragmentation, authentic standard, literature precedent, GNPS molecular networking, and/or temporal correlation, where applicable.

Analyze the metabolic transformation of EGCG in vivo using UHPLC-Q-Exactive Orbitrap-MS to reveal its dynamic changes within the body. A total of 106 fecal samples were available for LC–MS-based metabolomic analysis (CK, saline control; RE, EGCG gavage). Of the 14 scheduled samples that were unavailable for analysis, five could not be collected at the corresponding time points, whereas nine yielded insufficient fecal material for LC–MS sample preparation. Analysis of the 106 available samples yielded 14,931 annotated metabolite-related features (MRFs) in both negative and positive ion modes (Table S9). As shown in Fig. 6B, PCA analysis classified the metabolites into three groups: CK-0 h to 6 h and RE-0 h to 8 h (green ellipse), CK-8 h to 24 h and RE-24 h (red ellipse), and RE-10 h to 12 h (blue ellipse). This result indicates that EGCG undergoes three key metabolic phases within the first 24 h in rats. To further investigate the metabolic transformation of EGCG in vivo, 683 EGCG-associated candidate MRFs were selected from the 14,931 annotated MRFs by chemical-class filtering. These included 167 benzoic acids and derivatives, 6 hydrolyzable tannins, and 510 flavonoid-related MRFs, comprising 43 flavans, 12 flavones, 9 isoflavans, and 446 other flavonoids. PCA revealed that the 683 MRFs were clearly clustered into three groups: CK-0 h to 24 h, RE-0 h to 4 h, and RE-24 h formed one cluster; RE-6 h to 8 h formed another; and RE-10 h to 12 h with RE-8 h-7/8/9 formed the third (Fig. 6C). The results indicated that the major intestinal transit phase of EGCG in SD rats primarily occurs between 6 and 12 h post-administration, with most EGCG and its metabolites excreted by 24 h.

Based on the criteria of VIP > 1 and mixed-effects model-derived FDR-adjusted P < 0.05, 59 differential MRFs were further identified. The 59 MRFs were considered as core metabolites potentially associated with EGCG biotransformation (Table S10). The OPLS-DA models showed satisfactory goodness of fit and predictive performance, and the 200-permutation tests indicated no obvious overfitting (Table S16). The pathways in Fig. 6D and E are proposed on the basis of metabolite structural annotation and temporal behavior, together with literature precedent where available. The evidence supporting each reaction step is listed in Table S13.

Among these core MRFs, 13 were identified as flavans (Table 1). ME1, with a retention time of 4.805 min, exhibited a quasimolecular ion peak at *m/z* 459.092 (M + H, C_22_H_18_O_11_) in positive ion mode, which was consistent with the mass spectrum of EGCG. The major fragmentation ions of ME1 were observed at *m/z* 139.037 and *m/z* 289.068. The remaining 12 flavans can be categorized into four groups (Fig. 6D). Among them, ME2 was identified as a high-molecular-mass EGCG-associated product (group 1). EGCG is primarily converted into small-molecule compounds through enzymatic reactions in the body. However, the presence of ME2 in rat feces suggested that EGCG may undergo structural transformations in the rat intestine, leading to the formation of higher-molecular-mass metabolites. Additionally, ME3, ME4, ME5, ME6, ME7, and ME8 were monomeric catechins and their derivatives, centered around the catechin backbone (group 2). These compounds represent the fundamental structural units of tea polyphenols and the initial stages of EGCG metabolism. In addition, ME9, ME10, and ME11 were putatively annotated as theaflavin-related compounds (group 3) based on their accurate masses and MS/MS fragmentation characteristics. The occurrence of these theaflavin-related compounds in rat feces following EGCG administration suggests that oxidative transformations of catechin-related structures may occur in the intestinal environment, potentially involving both non-enzymatic reactions and gut microbial interactions. Moreover, ME12 and ME13 can be classified as a fourth group of flavans showing structural modifications consistent with possible enzymatic transformation. ME12 is likely a downstream product of catechin metabolism mediated by host enzymes or gut microbiota. ME13 is a dimethylated product of catechin, a modification that may alter its polarity, increase stability, and influence its metabolic kinetics. These enzymatically modified catechins may regulate their stability, bioactivity, bioavailability, and metabolic kinetics in the intestinal microenvironment, thereby impacting their biological effects on host health. These results reveal the potential metabolic conversion pathway of EGCG in the rat intestine, which may reflect contributions from host enzymatic processes, gut microbial metabolism, and non-enzymatic reactions.

**Table 1:** 

ID	MRF ID	Retention time (min)	Ion mode	m/z	Formula	Adducts	Metabolites
ME1	MRF 11187	4.805	pos	459.092	C_22_H_18_O_11_	M + H, M + NH_4_, M + H-H_2_O	Epigallocatechin Gallate
ME2	MRF 11245	4.805	pos	917.176	C_44_H_36_O_22_	M + H, M + Na, M + NH_4_	Assamicain A
ME3	MRF 11185	3.335	neg	305.064	C_15_H_14_O_7_	M-H, 2M-H	Epigallocatechin
ME4	MRF 11165	4.709	pos	291.086	C_15_H_14_O_6_	M + H-H_2_O, M + H	Epicatechin
ME5	MRF 11249	4.716	neg	289.07	C_15_H_14_O_6_	M-H, M + FA-H, 2M-H	(±)-Catechin
ME6	MRF 11195	3.297	pos	307.081	C_15_H_14_O_7_	M + H-H_2_O, M + H	(+)-Gallocatechin
ME7	MRF 11213	1.361	pos	307.081	C_15_H_14_O_7_	M + H, M + Na, M + H-H_2_O	4-Gallocatechol
ME8	MRF 11182	4.897	pos	305.101	C_16_H_16_O_6_	M + H	4-methyl-epicatechin
ME9	MRF 11398	5.019	neg	913.147	C_43_H_32_O_20_	M + FA-H	Theaflavin-3,3′-digallate
ME10	MRF 11550	4.860	neg	761.135	C_36_H_28_O_16_	M-H_2_O-H, M + FA-H	Isoneotheaflavin 3-O-gallate
ME11	MRF 11352	5.402	pos	581.094	C_28_H_20_O_14_	M + H, M + NH_4_	Epitheaflavic acid 3′-gallate
ME12	MRF 11229	4.835	pos	321.096	C_16_H_16_O_7_	M + H-H_2_O, M + H	Ourateacatechin
ME13	MRF 11215	5.282	pos	487.122	C_24_H_22_O_11_	M + H, M + Na	4′-Methyl-(−)-epigallocatechin 3-(4-methyl-gallate)
ME14	MRF 212	4.546	pos	309.096	C_15_H_16_O_7_	M + H, M + Na, M + H-H_2_O	4R,5R,6S-Trihydroxy-2-hydroxymethyl-2-cyclohexen-1-one 6-(2-hydroxy-6-methylbenzoate)
ME15	MRF 218	5.469	pos	553.096	C_27_H_20_O_13_	M + H	Epitheaflagallin 3-O-gallate

In addition, 167 GA-related benzoic acids and derivatives were identified in rat feces, though their relative abundances were generally low. Among them, only 4R,5R,6S-trihydroxy-2-hydroxymethyl-2-cyclohexen-1-one 6-(2-hydroxy-6-methylbenzoate) (ME14) and epitheaflagallin 3-O-gallate (ME15) had VIP > 1 and *P* < 0.05 (Table 1 and Fig. 6E). These two compounds were likely the major metabolic transformation products of GA in rat gut. Moreover, 43 flavonoids with VIP > 1 and *P* < 0.05 were identified (Table S10), potentially originating from EGCG transformations involving enzymatic modifications such as methylation, hydroxylation, and dehydroxylation. These modifications not only enhance the solubility or hydrophobicity of the compounds but may also influence their biological activity and metabolic stability.

### 3.4 Metabolic transformation of EGCG by rat gut microbiota under *in vitro* anaerobic fermentation

To explore the biotransformation of EGCG by rat gut microbiota, we conducted *in vitro* anaerobic fermentation. UPLC was employed to quantify EGCG levels in co-fermentation samples with gut microbiota under anaerobic conditions (AFE) at 0, 2, 4, 8, 12, 24, 36, 48, 60, and 72 h. In all three concentration gradients we established, EGCG levels in all AFE groups showed a sustained decline over time. This finding indicates that the rat gut microbiota significantly reduced EGCG levels (Fig. 7A–C). Although rat gut microbiota exhibited the ability to degrade EGCG *in vitro*, they unexpectedly failed to achieve complete degradation. Additionally, untargeted metabolomics analysis was employed to reveal the metabolic profiles of AFE (1.0 mg/mL EGCG) groups at different fermentation times. PCA and HCA analyses showed that the AFE samples from different fermentation times clustered together, with the AFE samples from 24 h, 36 h, 48 h, 60 h, and 72 h not being clearly distinguishable (Figure S1A and B). Among the 5942 identified compounds (anaerobic fermentation metabolites, AFM, Table S11), only 9 flavans were detected, including (−)-epigallocatechin gallate, epigallocatechin, (±)-catechin, epicatechin, assamicain A, leucocyanidin, 4′-methylepicatechin 5-sulfate, epicatechin 4′-sulfate, and epicatechin 7-sulfate (Table S12). These results indicate that rat gut microbiota primarily convert EGCG into monomeric catechins *in vitro*, accompanied by the detection of several sulfated catechin-related derivatives. Interestingly, seven of the nine metabolites mentioned above were also present in in vitro fungal fermentation, including (−)-Epigallocatechin gallate, EPIGALLOCATECHIN, (±)-Catechin, Epicatechin, Leucocyanidin, Epicatechin 4′-sulfate, and Epicatechin 7-sulfate. Moreover, the feature annotated as Epicatechin 4′-sulfate exhibited similar concentration trends both in vivo and *in vitro*, further supporting its association with EGCG-related intestinal biotransformation. Notably, the number of EGCG-associated metabolites detected *in vitro* was substantially lower than that observed in vivo. This difference may reflect the combined influences of the digestive environment, pH, host enzymatic activity, and the overall gut microbial community.

**Fig. 7:**
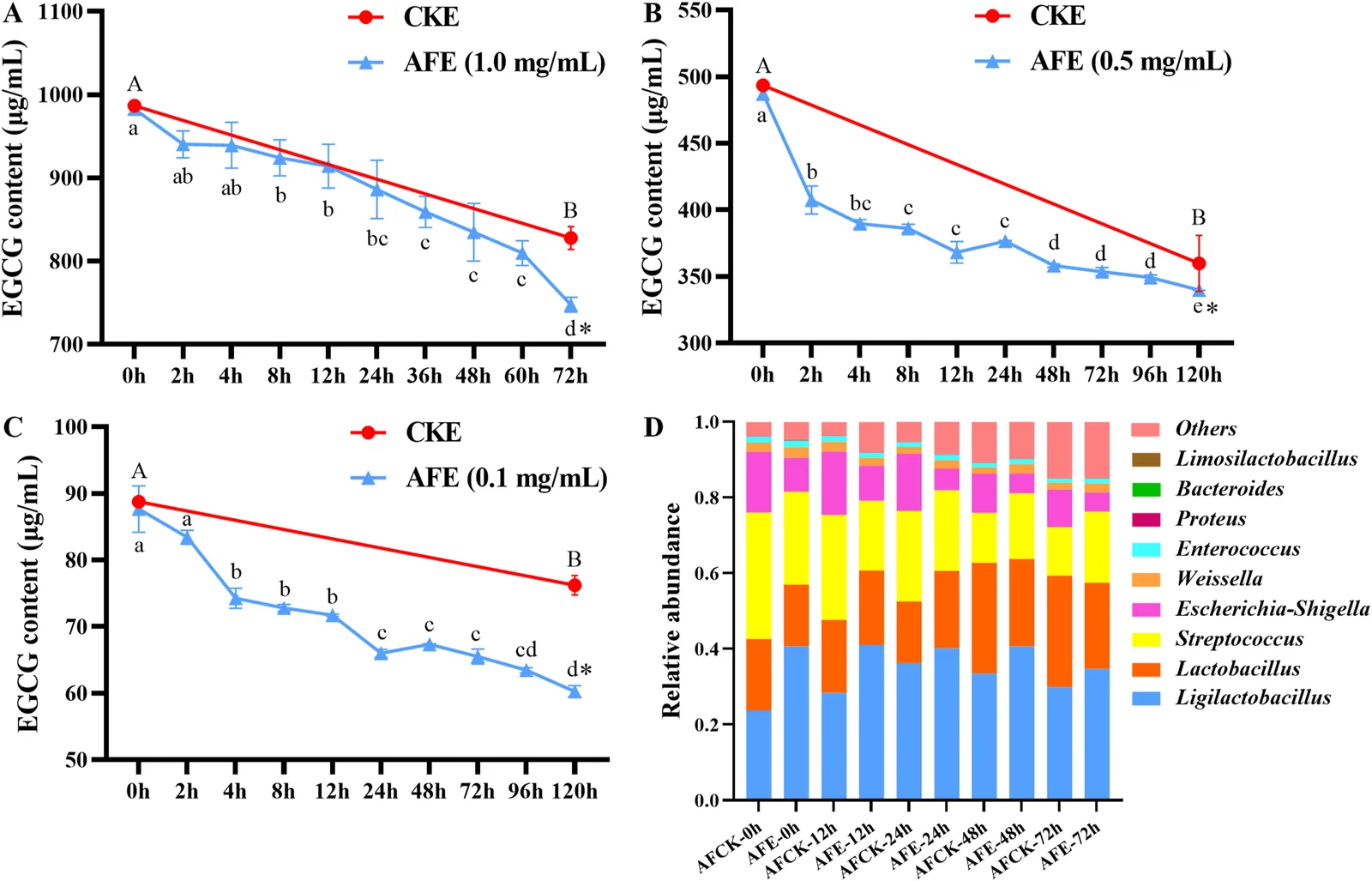
Metabolic transformation of EGCG by rat gut microbiota under *in vitro* anaerobic culture. (A-C) Changes in EGCG content in anaerobic co-fermentation samples with different EGCG concentrations (1.0 mg/mL EGCG, A; 0.5 mg/mL EGCG, B; 0.1 mg/mL EGCG, C) and gut microbiota (AFE) at different time points, detected by ultra-performance liquid chromatography (UPLC) analysis. CKE represents the medium control with EGCG. Significant differences in concentrations are marked with different uppercase (CKE) or lowercase (AFE) letters (one-way ANOVA; *P* < 0.05). (D) The top 10 most abundant gut microbial genera in the anaerobic fermentation control (without EGCG, AFCK) and AFE (1.0 mg/mL EGCG) groups at different fermentation times, detected by 16S rDNA sequencing.

To characterize temporal changes in gut microbial composition during EGCG biotransformation, 16S rDNA sequencing was performed on anaerobic fermentation control group (without EGCG, AFCK) and AFE (1.0 mg/mL EGCG) at different fermentation times (0, 12, 24, 48, and 72 h). Fig. 7D listed the top 10 most abundant gut microbial genera in the AFCK and AFE groups. The gut microbial composition in the AFCK and AFE groups was similar, with *Ligilactobacillus*, *Lactobacillus*, *Streptococcus*, and *Escherichia-Shigella* being the dominant genera. With the extension of fermentation time, the relative abundance of *Ligilactobacillus* and *Lactobacillus* increased in both AFCK and AFE groups, while the relative abundance of *Streptococcus* and *Escherichia-Shigella* decreased. These temporal abundance changes were observed alongside EGCG biotransformation during *in vitro* fermentation.

To further quantify the relationships between microbial composition and EGCG biotransformation, exploratory Spearman correlation analysis was performed between the relative abundances of the four dominant bacterial genera and EGCG or EGCG-associated metabolites using matched mean values at the five 16S rDNA sampling time points (0, 12, 24, 48, and 72 h; n = 5). Several strong temporal associations were observed. Lactobacillus abundance was inversely associated with targeted EGCG concentration (Spearman ρ = −0.90, FDR-adjusted P = 0.3704), whereas Escherichia-Shigella showed a positive association with EGCG (ρ = 0.90, FDR-adjusted P = 0.3704). The strongest observed association was between Escherichia-Shigella and 4′-methylepicatechin 5-sulfate (ρ = 1.00, FDR-adjusted P = 0.3704). However, none of the taxon–metabolite correlations remained statistically significant after Benjamini–Hochberg FDR correction (all adjusted P > 0.05). Therefore, these relationships should be interpreted as exploratory temporal co-variation rather than evidence of direct microbial metabolism. The complete correlation statistics are provided in Table S17.

## 4 Discussion

Glucuronidation, methylation, or ring cleavage products of EGCG typically reduce its biological activity. However, certain downstream metabolites generated through biotransformation exhibit enhanced stability, bioavailability, and even superior biological activity (Lambert et al., 2007; López de Lacey et al., 2014; S. Zhang et al., 2024). This facilitates EGCG’s more effective execution of its biological functions (Meng et al., 2002). Microbial-driven biotransformation of catechins is a key strategy to enhance its bioavailability, improve structural stability, and mitigate potential toxicity at high doses, including transformations by intestinal flora and fungal fermentation (Lai et al., 2020; C. Liu et al., 2024; Liu et al., 2023). As predominant fungi in dark tea fermentation, *A. cristatus* and *A. niger* drive the biotransformation of catechins, imparting distinct physiological effects such as hypoglycemic, lipid-lowering, and anti-obesity properties, which differentiate dark tea from other tea types (Cheng et al., 2025; Huang et al., 2024; Zhu et al., 2020). However, the biotransformation of EGCG by tea-derived fungi and gut microbiota remains poorly understood. This study combines targeted and untargeted metabolomics to elucidate the metabolic fate of EGCG mediated by *A. cristatus* and *A. niger*, offering comprehensive metabolic profiles of dark tea fermentation involving these fungi and investigating the metabolic transformation of EGCG by gut microbiota in both *in vitro* and in vivo experiments.

### 4.1 Distinct metabolic conversion of EGCG by *A. cristatus* and *A. niger*

When *A. cristatus* and *A. niger* were fermented with EGCG, *A. cristatus* achieved complete degradation of EGCG within 12 h, whereas *A. niger* required 24 h. This aligns with previous reports that *A. niger* requires at least 24 h to completely degrade EGCG (Liu et al., 2023; Qin et al., 2012). Kinetic analysis further showed that the apparent EGCG disappearance rate constant for *A. cristatus* was approximately 3.05-fold that for *A. niger*, accompanied by a markedly shorter half-life (Table S14). These results quantitatively support faster EGCG disappearance during *A. cristatus* fermentation under the present liquid-culture conditions. Notably, the opposite trend was observed in the dark-tea matrix, suggesting that the relative EGCG transformation kinetics of the two fungi are substrate dependent. Biologically, this reversal indicates that the EGCG-transforming capacity of these fungi is context dependent and may reflect interactions between species-specific metabolic activities and the surrounding substrate environment. It is well-established that under microbial activity, the ester bond in EGCG is first hydrolyzed, releasing EGC and GA (Liu et al., 2022). Simultaneously, in both the ACF and ANF groups, epigallocatechin-(4*β*→8)-epicatechin 3,3′-digallate was identified as an early-stage EGCG-associated metabolite. Additionally, more complex polyflavonoids, such as samarangenin A, were identified among the early-stage EGCG-associated metabolites in the ACF group. These findings indicate that during the early stages of fungal fermentation, the metabolic transformation of EGCG involves not only degradation but also complex polymerization processes. Similarly to EGCG, the degradation rate in the ACF group was significantly faster than in the ANF group. This difference in degradation efficiency may be attributed to the inhibition of *A*. *niger* conidial aggregation by EGCG, which suppressed the expression of chitinases and chitin synthases, thereby affecting the structure of the hyphal wall and interfering with the normal growth of *A. niger* (Chen et al., 2019; Liu et al., 2023; Sun et al., 2018).

During mid-fermentation, *A. cristatus* and *A. niger* produced several identical EGCG-related metabolites including demethyltorosaflavone D, theasinensin B, oolongtheanin, theaflavin 3,3′-digallate, prodelphinidin A2 3′-gallate, and isoneotheaflavin 3-O-gallate (Fig. 2, Fig. 3B). Among these, demethyltorosaflavone D is a flavonoid compound that has demonstrated potential anti-diabetic and anti-aging properties (Aon-Im et al., 2024; Huong et al., 2023); Theasinensin B exhibits antihypertensive effects and inhibits alpha-glucosidase activity (Li et al., 2013; Tao et al., 2020); Oolongtheanin is effective in lowering cholesterol (Ogawa et al., 2016); Theaflavin 3,3′-digallate demonstrates strong antiviral and anti-constipation properties (Qin et al., 2023; Wu et al., 2024). Additionally, morelosin with anti-hyperglycemic activity and theasinensin A with lipid-lowering effects were identified in ACF (Tamaru et al., 2013; Yang et al., 2022). In addition, tragopogonic acid, theaflavin-3-gallate, 3′-galloylprodelphinidin B2, (E)-N-(3-(3-(4-fluorophenoxy)phenyl)-1-(R,S)-methylprop-2-enyl)-N-hydroxyurea, and salicyl-6-hydroxy-2-cyclohexene-on-oyl were identified as characteristic mid-stage EGCG-associated metabolites during *A. niger* fermentation. Overall, several of the EGCG-related compounds detected during fungal fermentation have previously been reported to exhibit biological activities, providing literature-based context for their potential functional relevance.

In the late fermentation stage, the metabolic conversion of EGCG and GA exhibited distinct changes under the influence of *A. cristatus* and *A. niger*. 3-Hydroxyflavone (Met, 1893) is one of the late-stage EGCG-associated metabolites in ACF with high abundance (Table S2). 3-Hydroxyflavone has previously been reported as a biotransformation metabolite of flavonoids mediated by *A. niger* (Cao et al., 2015). However, in this study, the abundance of 3-hydroxyflavone produced by *A. cristatus* in the later fermentation stage exceeded that of *A. niger* by over 100-fold (Fig. 2B and Table S1). For 3-Hydroxyflavone, *A. cristatus* exhibits a higher conversion efficiency. Additionally, several relatively abundant late-stage EGCG-associated metabolic features were observed in ACF, with database-assisted annotations corresponding to dihydroxyphaseic acid, lidoflazine, camonagrel, butralin, ellagic acid, and pioglitazone. These features further illustrate the chemical diversity associated with the late-stage transformation of EGCG and GA during *A. cristatus* fermentation and provide candidates for further structural and functional characterization. Since *A. niger* exhibits a relatively slow degradation rate for EGCG, we define the 36-h fermentation period of ANF as the mid-to-late stage of the metabolic process. At this point, the relative abundance of several flavonoid compounds peaks, including quercetin 3-(6″-caffeoylgalactoside), 5,7,3′,4′,5′-pentahydroxyflavanone, isotheaflavin, fuzhuanins A, and prodelphinidin A1 (Fig. 3B). These compounds were therefore considered putative EGCG-associated metabolites during *A. niger* fermentation. This result is somewhat inconsistent with previously reported biotransformation products of EGCG by *A. niger* (RAF106), and the differences may be attributed to variations in fungal strains and culture medium (T. Liu et al., 2022). In summary, the identification of key EGCG-related metabolites generated at different time points during these metabolic processes contributes to understanding the biotransformation of EGCG by *A. cristatus* and *A. niger*.

### 4.2 Metabolic differences between *A. cristatus* and *A. niger* in dark tea matrix fermentation

Due to differences in the biotransformation of EGCG by *A. cristatus* and *A. niger*, both can convert EGCG into distinct compounds with health benefits. An increasing number of studies are focusing on dark tea fermented using a single microbial community (An et al., 2021; Zhu et al., 2020). Unlike microbial fermentation using EGCG as the substrate, dark tea contains a wide range of secondary metabolites. The metabolic transformation of EGCG by *A. cristatus* and *A. niger* exhibits significant differences when performed on tea matrix. Surprisingly, the degradation rate of EGCG in ACFT was lower than that in ANFT. This result contradicts our previous observation that the degradation rate of EGCG in ACF was higher than that in ANF. This finding aligns with Zeng et al. (2023), who reported that after 5 days of fermentation, the relative EGCG content in dark tea fermented by *A. cristatus* was higher than that in the *A. neoniger* group. This suggests that the substrate plays a critical role in shaping the metabolic activity of *A. cristatus* and *A. niger*. It should also be noted that the tea matrix was autoclaved at 121°C for 15 min before fungal inoculation. Such moist-heat treatment may induce changes in catechins, polyphenols, proteins, and their interactions, thereby partially reshaping the chemical environment of the tea matrix. These heat-induced changes may also contribute to the substrate-dependent metabolic behavior of *A. cristatus* and *A. niger*. As untreated raw tea and immediately post-autoclaved tea were not directly compared in the present study, the potential influence of autoclaving should be taken into consideration when extrapolating these findings to natural dark-tea fermentation. Interactions between the substrate and fungus may occur, such as high concentrations of EGCG inhibiting the activity of *A. niger* without exerting the same effect on *A. cristatus*. Additionally, the tea leaf matrix may guide *A. cristatus* toward alternative metabolic pathways, thereby reducing its efficiency in degrading EGCG. Differences in enzyme activity and the influence of environmental factors arising from distinct substrates may both impact the metabolic processes of *A. cristatus* and *A. niger*. From a practical perspective, these substrate-dependent differences suggest that fungal strain selection and fermentation conditions may be used to modulate the extent of EGCG depletion and the formation of catechin-related derivatives in dark tea. These findings therefore provide a basis for more targeted optimization of controlled dark tea fermentation, although further validation under natural or industrial fermentation conditions is required. This substrate dependence was also reflected in the EGCG-associated metabolic profiles. In the liquid system, *A. cristatus* and *A. niger* shared several metabolites, including EGC, epigallocatechin-(4β→8)-epicatechin 3,3′-digallate, theasinensin B, oolongtheanin, prodelphinidin A2 3′-gallate, and isoneotheaflavin 3-O-gallate, suggesting partially conserved hydrolysis, oxidation, and coupling reactions. However, their downstream profiles differed, with pronounced accumulation of 3-hydroxyflavone in *A. cristatus* fermentation and a broader range of late-stage flavonoid-related metabolites in *A. niger* fermentation. Similar overlap and divergence were also observed in the dark-tea matrix, where several catechin-related metabolites were shared between ACFT and ANFT, while each fungus exhibited distinct EGCG-associated metabolites and metabolic-network relationships. Together with the reversed EGCG disappearance rates between the two systems, these results indicate that the two fungi share some basic transformation routes but redirect EGCG metabolism differently depending on the substrate environment.

In the dark tea matrix, catechins can be degraded or react with other compounds under the enzymatic action of *A. cristatus* or other *Aspergillus* spp., forming new compounds such as non-galloylated catechins (e.g., EGC and EC) or flavan-3-ol B-ring fission analogs like teadenols and fuzhuanins (Wulandari et al., 2011; Xie et al., 2020). These compounds may possess more stable structures and higher bioavailability compared to EGCG. Previous studies have reported the non-volatile metabolic profiles of dark tea fermented solely by *A. cristatus* or *A. niger* (Xiao et al., 2022; Zeng et al., 2023). Xiao et al. (2022) identified 574 differential metabolites at different fermentation stages in *A. cristatus* under solid-state fermented dark tea using UPLC-QQQ-MS/MS. Dynamical changes in metabolites of dark tea fermented by *A. cristatus* and *A. neoniger* were analyzed through temporal clustering analysis using UPLC-QTOF-MS/M by Zeng et al. (2023). These researches provide a rich data foundation for understanding the metabolic profiles of dark tea fermented by *A. cristatus* and *A. niger*. In this study, a total of 5144 metabolites in ACFT and ANFT at different fermentation stages are identified using high-throughput UHPLC-Q-Exactive Orbitrap-MS-based metabolomics. The core metabolic modules associated with EGCG biotransformation are identified using WGCNA. Among these, (±)-catechin (MFT 3211), epigallocatechin (MFT 3214), (−)-epiafzelechin 3-gallate (MFT 3255), (±)-taxifolin (MFT 3265), epigallocatechin 3-O-caffeate (MFT 3287), epiafzelechin 3-O-gallate (MFT 3316), taxifolin (MFT 3332), neotheaflavin (MFT 3534), epicatechin 3-O-(4-methylgallate) (MFT 3617), butin (MFT 3647), (−)-epigallocatechin 3-(3-methyl-gallate) (MFT 3688), isoneotheaflavin 3-O-gallate (MFT 3236), theaflavic acid (MFT 3261), trans-3,3′,4′,5,5′,7-hexahydroxyflavanone (MFT 3398), and (−)-epigallocatechin 3,3′-di-gallate (MFT 3621) are considered as core flavans involved in EGCG biotransformation in dark tea fermented by *A. cristatus* (Tables S5 and S6). In addition, distinct core flavans associated with EGCG and GA are identified in *A. niger*-fermented dark tea, differing from those in ACFT (Tables S7 and S8).

By linking the temporal changes in EGCG with fungus-associated metabolic modules and molecular networks, this study extends previous metabolomic descriptions of dark tea fermentation and provides a more integrated view of microbial-mediated catechin transformation. In particular, our findings indicate that EGCG transformation during dark tea fermentation is a strain- and matrix-dependent process rather than a simple, uniform degradation pathway. Furthermore, we discovered several unknown compounds structurally similar to major catechins such as EGCG, EGC, and EC using GNPS analysis. These findings provide a valuable basis for future isotope-tracing studies aimed at confirming the microbial biotransformation pathways of these key catechins.

### 4.3 Differences in EGCG-associated metabolic profiles between the in vivo rat model and the *in vitro* gut microbiota system

Catechins exhibit low oral bioavailability, primarily undergoing metabolism and absorption in the colon. However, their transformation within the colon enables diversification of their biological activity. In this study, EGCG levels in rat feces were analyzed after oral administration of EGCG. EGCG appeared in feces 6 h post-administration, peaked at 10 h, and declined thereafter, becoming nearly undetectable at 24 h. This result slightly differs from previous findings. Kohri et al. (2001) detected EGCG in rat feces 8–12 h after gavage, with peak levels observed at 12–24 h, and detectable EGCG persisting until 48–72 h. A possible explanation for this discrepancy is the use of different rat strains: we employed Sprague-Dawley (SD) rats, whereas Kohri et al. (2001) used Wistar rats, which may exhibit significant differences in EGCG metabolism. Based on the changes in flavan compounds in rat feces, we proposed a potential metabolic pathway of EGCG in rats (Fig. 6D). In addition to EGCG derivatives generated through enzymatic oxidation, cleavage, and modification, we identified theaflavins and their derivatives potentially associated with oxidative transformation. These findings indicate that the biotransformation of EGCG in the rat gut may involve both microbial enzyme-mediated reactions and non-enzymatic processes influenced by the internal environment. This finding enhances our understanding of EGCG metabolism and its health-related effects in vivo.

Additionally, we found that the anaerobic co-fermentation of EGCG with rat gut microbiota *in vitro* exhibited limited EGCG degradation capacity. After 72 h of fermentation, only small amounts of initial EGCG metabolites, such as EGC, EC and C, together with several sulfated catechin-related features, were detected (Table S12). The marked discrepancy between the in vivo and *in vitro* results suggests that the broader EGCG-associated metabolic changes observed in vivo cannot be explained by fecal microbiota alone. Host enzymatic reactions and intestinal absorption may contribute to this difference, while gastrointestinal transit exposes EGCG to distinct intestinal compartments rather than the relatively uniform conditions of an *in vitro* culture. In addition, dynamic pH, oxygen/redox conditions, bile, dietary components, and mucus-associated microenvironments may influence EGCG stability, accessibility, non-enzymatic oxidation, and microbial interactions. Thus, the broader in vivo metabolic profile likely reflects the combined effects of gut microbial metabolism, host processes, and the gastrointestinal environment rather than microbiota alone.

Within the *in vitro* system, *Ligilactobacillus*, *Lactobacillus*, *Streptococcus*, and *Escherichia-Shigella* were among the dominant genera detected during *in vitro* anaerobic fermentation, and their temporal abundance patterns coincided with EGCG biotransformation. Previous studies have reported the ability of bacteria from the human intestinal microbiota, such as those from the *Butyrivibrio* and *Eubacterium* genera, to catalyze catechin C-ring fission and B-ring dihydroxylation (Chen and Yang, 2020; Marín et al., 2015; Wang et al., 2001). However, this study did not identify the specific *in vitro* biotransformation pathways of EGCG mediated by the gut microbiota of SD rats.

An important limitation of the present study is that isotope-labelled EGCG was not used for metabolic tracing. Therefore, although the metabolites described above were identified as EGCG-associated candidates based on their metabolomic profiles and structural annotations, their direct derivation from EGCG cannot be conclusively established. Depending on the experimental system, endogenous fungal metabolites, native tea constituents, and host-, diet-, or microbiota-derived compounds may change simultaneously with EGCG and may therefore contribute to the observed metabolic differences. Accordingly, the proposed EGCG metabolic pathways and downstream metabolites should be interpreted as putative rather than definitively confirmed EGCG-derived products. Future studies using stable-isotope-labelled EGCG, such as ^13C-labelled EGCG, combined with high-resolution tandem mass spectrometry will be necessary to trace the metabolic fate of EGCG and confirm precursor–product relationships. In addition, more suitable gut microbiota host donors should be selected for future *in vitro* EGCG biotransformation experiments.

## 5 Conclusion

In summary, the present study employed targeted and untargeted metabolomics to characterize EGCG biotransformation by tea-derived fungi and rat gut microbiota. EGCG disappeared faster during *A. cristatus* fermentation in liquid culture, whereas *A. niger* caused faster EGCG depletion in dark tea, demonstrating that the relative transformation kinetics of the two fungi were substrate dependent. Untargeted metabolomics highlighted key EGCG-associated metabolites at different fermentation stages, with distinct clustering of metabolites during fermentation. *A. cristatus* demonstrated a more extensive and complex metabolic network for EGCG and GA, involving diverse chemical modifications such as oxidation, cleavage, and hydroxylation. In contrast, *A. niger* fermentation resulted in fewer but unique derivatives, including late-stage flavonoid metabolites. Metabolic profiling of dark tea fermented with these fungi identified structurally diverse EGCG-associated metabolites and highlighted fungus-associated differences in metabolite profiles. In vivo experiments further showed extensive changes in EGCG-associated metabolites, including polymeric and structurally modified catechin-related features, with significant changes occurring between 6 and 12 h post-administration. However, *in vitro* anaerobic cultures of rat gut microbiota displayed limited degradation capabilities, suggesting that the broader in vivo transformation may reflect the combined contributions of gut microbial activity, host enzymatic processes, intestinal absorption and transit, and digestive system-specific conditions. For dark tea fermentation specifically, the substrate-dependent kinetics and fungus-associated metabolite patterns identified here broaden our understanding of how dominant fungi contribute to catechin remodeling during fermentation. These findings provide valuable insights into EGCG metabolism by fungi and gut microbiota, offering a foundation for optimizing tea fermentation processes and exploring the bioavailability and health benefits of catechins. Future studies should further distinguish the microbial, host enzymatic, and environmental contributions to EGCG biotransformation.

## CRediT authorship contribution statement

Zexin An: Writing – original draft, Visualization. Rongrong Ma: Investigation. Xiangxiang Huang: Investigation. Yuhang Zhang: Formal analysis. Zhonghua Liu: Methodology. Jianan Huang: Methodology. Kunbo Wang: Validation, Conceptualization. Mingzhi Zhu: Writing–review & editing, Project administration, Methodology. All authors reviewed the manuscript and approved the final version of the manuscript.

## Declaration of competing interest

The authors declare that they have no known competing financial interests or personal relationships that could have appeared to influence the work reported in this paper.
