# Supplementary material for: Aspergillus species and gut microbiota-mediated biotransformation of epigallocatechin gallate: From dark tea fermentation to intestinal metabolism
Source: Curr Res Food Sci. 2026 Oct 8;13:101587. doi: 10.1016/j.crfs.2026.101587 (PMC13717108; doi:10.1016/j.crfs.2026.101587)
Supplement: Supplementary file 1 [file mmc2.pdf]

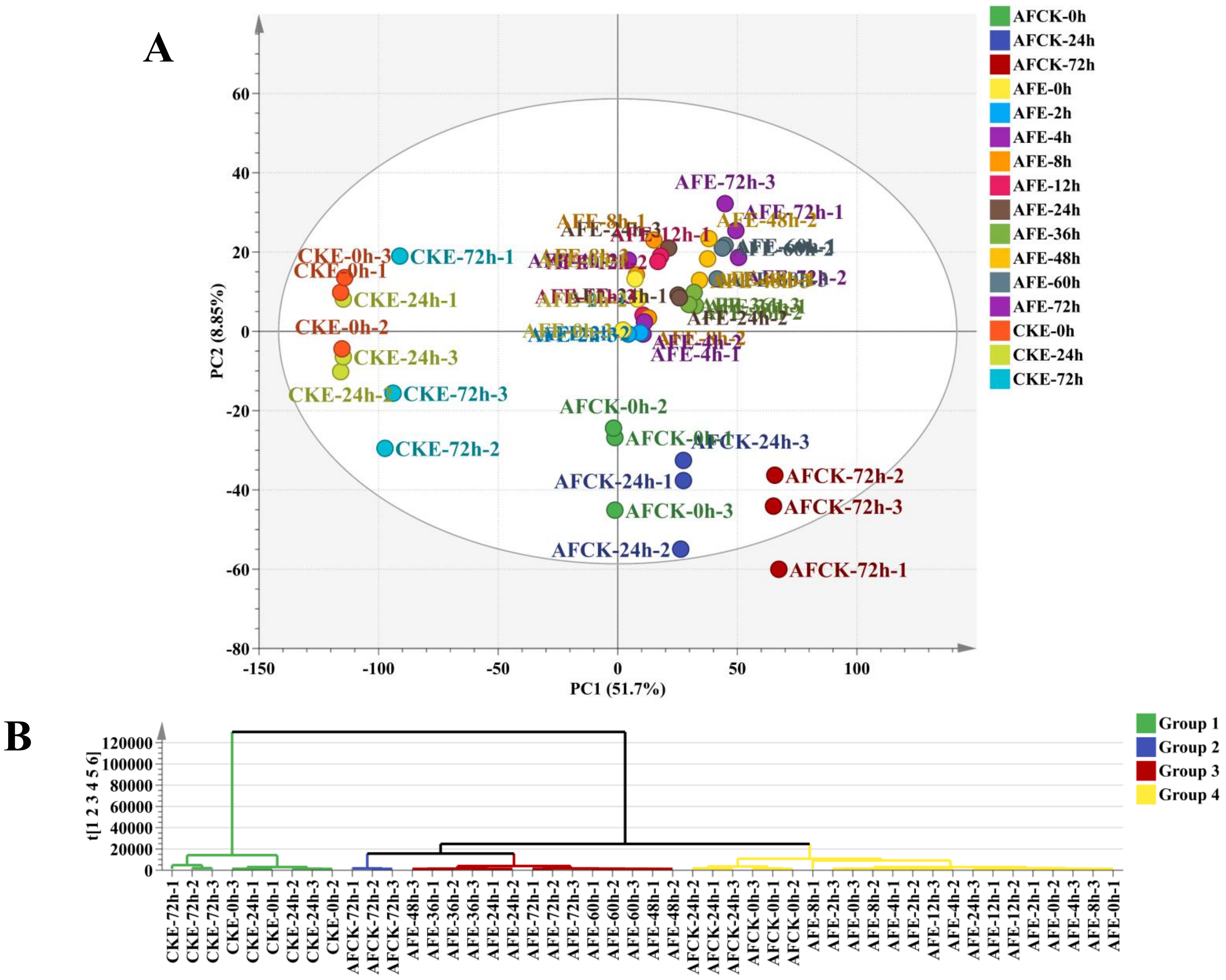
A
PC1 (51.7%)
PC2 (8.85%)
AFCK-0h
AFCK-24h
AFCK-72h
AFE-0h
AFE-2h
AFE-4h
AFE-8h
AFE-12h
AFE-24h
AFE-36h
AFE-48h
AFE-60h
AFE-72h
CKE-0h
CKE-24h
CKE-72h
B
t[1 2 3 4 5 6]
Group 1
Group 2
Group 3
Group 4

Figure S1
